# Distinct roles for primate caudate dopamine D1 and D2 receptors in visual discrimination learning revealed using shRNA knockdown

**DOI:** 10.1038/srep35809

**Published:** 2016-11-02

**Authors:** Masafumi Takaji, Atsushi Takemoto, Chihiro Yokoyama, Akiya Watakabe, Hiroaki Mizukami, Keiya Ozawa, Hirotaka Onoe, Katsuki Nakamura, Tetsuo Yamamori

**Affiliations:** 1 RIKEN, Brain Science Institute, Saitama, Japan; 2 Primate Research Institute, Kyoto University, Aichi, Japan; 3 RIKEN, Center for Life Science Technologies, Hyogo, Japan; 4 Jichi Medical University, Tochigi, Japan

## Abstract

The striatum plays important motor, associative and cognitive roles in brain functions. However, the rodent dorsolateral (the primate putamen) and dorsomedial (the primate caudate nucleus) striatum are not anatomically separated, making it difficult to distinguish their functions. By contrast, anatomical separation exists between the caudate nucleus and putamen in primates. Here, we successfully decreased dopamine D1 receptor (D1R) or D2R mRNA expression levels selectively in the marmoset caudate using shRNA knockdown techniques, as determined using positron emission tomography imaging with specific D1R and D2R ligands and postmortem *in situ* hybridization analysis. We then conducted a voxel-based correlation analysis between binding potential values of PET imaging and visual discrimination learning task performance in these genetically modified marmosets to find a critical role for the caudate D2R but no apparent role for the caudate D1R. This latter finding challenges the current understanding of the mechanisms underlying D1R activation in the caudate.

The basal ganglia consist of multiple nuclei, including the striatum^1^, which plays a major role in the control of motor actions and the malfunction leads to Parkinson disease^2^. Recent studies revealed that the striatum also plays multiple roles in motor, habitual, and cognitive functions^3^,^4^,^5^. Cell-type-specific transgenic mouse lines in combination with optogenetic approaches have demonstrated precise roles of specific neural circuits in striatal functions^6^,^7^. In the rodent striatum, data from the motor and sensory, associative, and anterior cingulate cortices are connected to the dorsolateral, dorsomedial, and ventral striatal regions, respectively^3^, as shown by local disruption of each region^8^,^9^. The results of a recent intact-brain analysis study, integrating an imaging technique that turns brain tissue transparent (CLARITY) with light sheet microscopy (CLARITY-optimized light-sheet microscopy), optogenetics, viral tracing, and fiber photometry, demonstrated dopamine subcircuits between the substantia nigra pars compacta (SNc) and DMS and dorsolateral striatum (DLS) in mice^10^. However, because the rodent dorsolateral and dorsomedial striatum (DLS and DMS) are not anatomically separated, it is difficult to distinguish their functions. In addition, the rodent frontal cortex, which consists of sensory motor, orbital, limbic, and cingulate cortices and interacts with the striatum, may be different from that in primates^11^,^12^,^13^. It has been suggested that the putamen and caudate nucleus play distinct roles^3^,^14^. For example, dopamine D2 receptors (D2R) in the caudate nucleus are suggested to function in the control of the cognitive switch in humans and marmosets^15^,^16^. In primates, the caudate, putamen, and ventral striatum are anatomically distinguished^17^. Motor pathways mainly exist in the putamen, whereas oculomotor and prefrontal circuits primarily occupy the caudate nucleus, and the limbic circuit is in the ventral striatum^2^,^18^. Therefore, the caudate nucleus or the putamen in the primate striatum could be selectively knocked down if a particular molecule could be effectively targeted in a region-specific manner.

Here, we directly tested this possibility using viral vector-mediated RNA interference^19^ in the marmoset. We selected D1R and D2R as the target molecules because they are the major dopamine receptors playing critical roles in striatal functions through activation and inhibition, respectively, of cortical-striatal-thalamic circuits^6^,^20^. The use of RNA targeting rather than pharmacological methods is advantageous because of the limited specificities of D1R and D2R agonists and antagonists. For example, even a highly selective antagonist for D2R retains some affinity for D3R and D4R^21^,^22^. The RNA targeting method also provides regional selectivity, effects that can be evaluated using positron emission tomography (PET) and postmortem *in situ* hybridization (ISH), and a long duration of action. In addition, because only the striatal neurons taking up the shRNA are affected, the presynaptic D2R on neurons projecting to the striatum are not. These advantageous features can be used to identify the unambiguous roles of striatal D1R and D2R in cognitive functions.

In the present study, we were able to distinguish the role of the D1R and D2R in the marmoset caudate nucleus, using shRNA-mediated specific knockdown of D1R or D2R mRNA. We found significant effects on D2R but no apparent phenotype on D1R in the caudate nucleus during visual discrimination learning.

## Results

### AAV-shRNAs efficiently knock down the D1R and D2R in marmoset caudate nucleus

We used viral vector-mediated shRNA targeting of D1R and D2R in the marmoset caudate nucleus to examine the effects of knocking down these receptors on the performance of a visual discrimination learning task in which the marmoset was required to select one of a pair of visual stimuli. We used adeno-associated virus (AAV) vectors because we found that the AAV vectors infect a wide region, and therefore are able to knock down the target RNA more efficiently than lentivirus vectors. To determine the shRNA that could most efficiently knock down D1R and D2R mRNA, we examined a series of shRNAs using an *in vitro* assay system. The most efficient shRNA achieved as high as 89% (for D1R) and 86% (for D2R) reductions in reporter expression levels using our assay system (Methods and Supplementary Fig. 1). To use shRNA sequences for *in vivo* experiments, the shRNA connected to the U6 promoter was integrated into an AAV2 (capsid) vector containing a separate transcriptional unit with the synapsin I promoter to express humanized renilla green fluorescent protein (hrGFP; Supplementary Fig. 2a).

**Figure 1:**
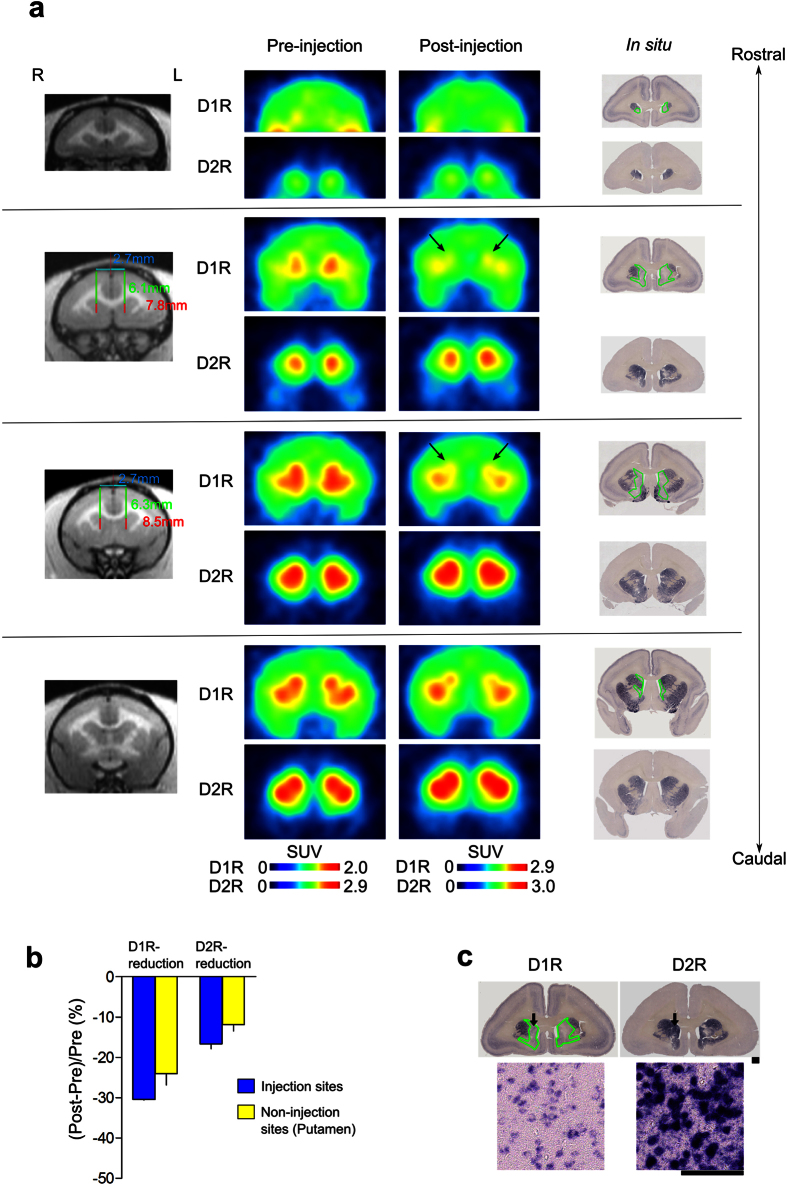
MR, PET, and *in situ* hybridization (ISH) images obtained in a marmoset with the D1R knocked down in the caudate nucleus. (**a**) Example images of a D1R KD marmoset (ID: M3, male, aged 2 years and 5 months at the time of the operation) are shown at four different coronal levels from rostral (top) to caudal (bottom) (see Supplementary Fig. 3 for another marmosets used for the experiment). The left panels are MR images showing the coordinates where the AAV-shRNA was injected. The middle panels are PET images comparing D1R (upper row) and D2R (lower row) ligand binding before and after AAV-shRNA injections. Arrows indicate regions where a significant reduction was observed. The right panel shows the ISH results. Regions outlined in green display significant reductions in mRNA expression for the D1R (upper row), with no change in D2R mRNA expression (lower row). (**b**) The graph shows a significant difference in the reductions between D1R and D2R BPnds (mean ± SD) in the injection sites (t(8) = 2.74, *P* = 0.025), with no significant difference in the reductions for the two receptor subtypes in the non-injection sites of the D1R KD marmoset (t(8) = 1.28, *P* = 0.238). (**c**) ISH results of D1Rs and D2Rs. The lower panel shows higher magnification images obtained in the areas indicated by the arrows. Scale bars: upper panel, 1 mm; lower panel, 0.1 mm.

**Figure 2:**
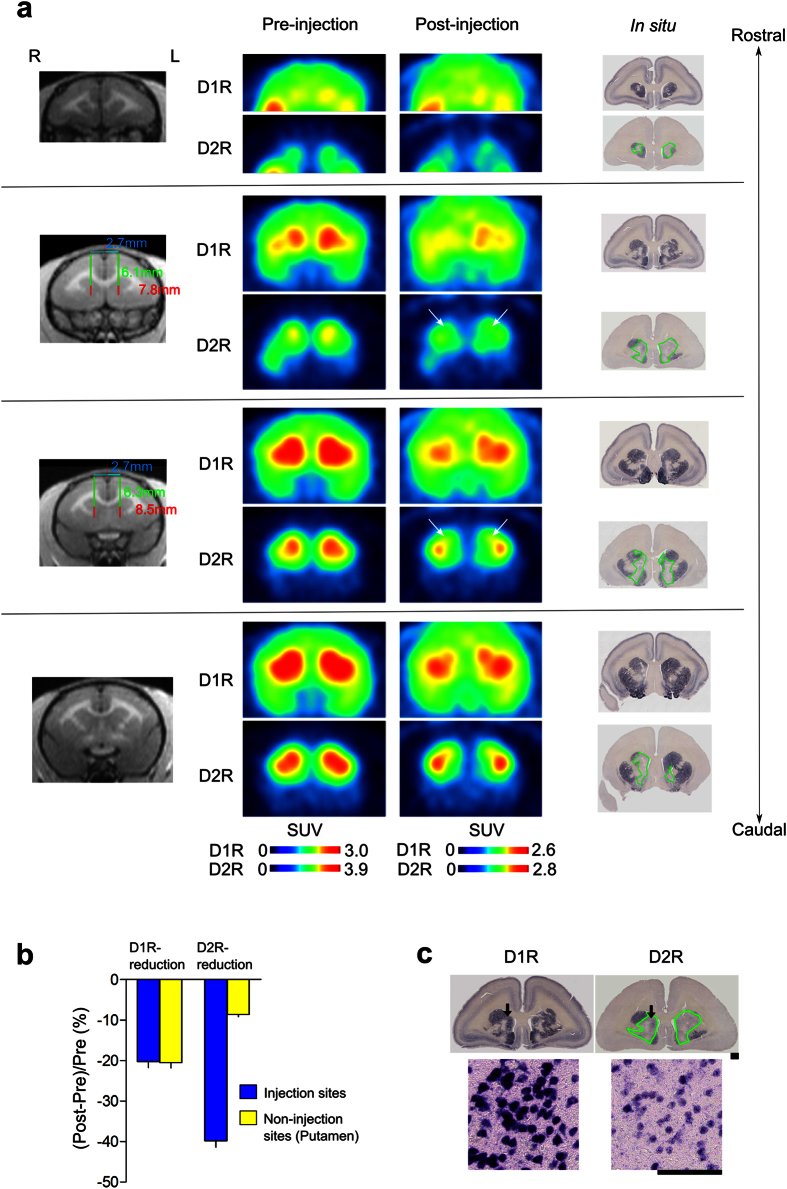
MR, PET, and *in situ* hybridization (ISH) images obtained in a marmoset with the D2R knocked down in the caudate nucleus. (**a**) Example images of a D2R KD marmoset (ID: M8, male, aged 2 years at the time of the operation) are shown as described in Fig. 1 (see Supplementary Fig. 3 for another marmosets used for the experiment). The right panel shows the ISH results, with regions outlined in green displaying significant reductions in mRNA expression for the D2R (lower row), with no change in D1R mRNA expression (upper row). (**b**) The graph shows a significant difference in the reductions between D1R and D2R BPnds (mean ± SD) in the injection sites (t(8) = −2.48, *P* = 0.038), with no significant difference in the reductions for the two receptor subtypes in the non-injection sites of the D2R KD marmoset (t(8) = 1.95, *P* = 0.087). (**c**) ISH results of D1Rs and D2Rs in the brain of the D2R KD marmoset. The lower panel shows higher magnification images of the areas indicated by the arrows. Scale bars: upper panel, 1 mm; lower panel, 0.1 mm.

We used PET to evaluate the expression levels of the caudate D1R and D2R proteins knocked down by shRNA *in vivo*. PET image analysis is useful for investigating relationships between receptor expression levels and behavior after receptor knockdown, as the animals do not need to be sacrificed to evaluate receptor binding activity. For stereotaxical injections, we measured the coordinates using magnetic resonance imaging (MRI; Supplementary Fig. 2b). The expression levels of D1R and D2R were measured as binding potentials (BPnds) by PET using ^11^C-SCH23390 and ^11^C-raclopride, respectively (Figs 1a and 2a). The quantitatively evaluated BPnd PET images were averaged for all animals before and after the injection of AAV2-shRNA-D1R or AAV2-shRNA-D2R (Figs 1b and 2b). Although D1R- and D2R-targeted shRNA administration affected the expression levels of D1R and D2R distant from the injection site in the putamen, the decreases in BPnds between D1R and D2R in the injected caudate nucleus were significantly different from those in the non-injection sites (Figs 1b and 2b).

The results of the ISH assay showed specific reductions in target gene mRNA expression levels in the injected caudate regions (Figs 1c and 2c, within the green outlined region). Injection of D1R-targeted shRNA decreased the level of D1R mRNA expression to background levels, while the level of D2R expression in these marmosets remained constant (Fig. 1c). Similarly, D2R-targeted shRNA decreased the level of D2R mRNA expression to background without altering D1R expression (Fig. 2c). A comparison of the results from PET and ISH analyses confirmed that the D1R and D2R mRNA expression levels were effectively decreased in the shRNA-injected regions (Figs 1 and 2). To summarize, as shown in Supplementary Fig. 3, we detected specific knockdown of the targeted dopamine receptor mRNAs in the medial caudate nucleus in all marmosets tested, despite the sample-to-sample variance among different injections.

### D2R KD marmosets show visual learning deficits

**Figure 3:**
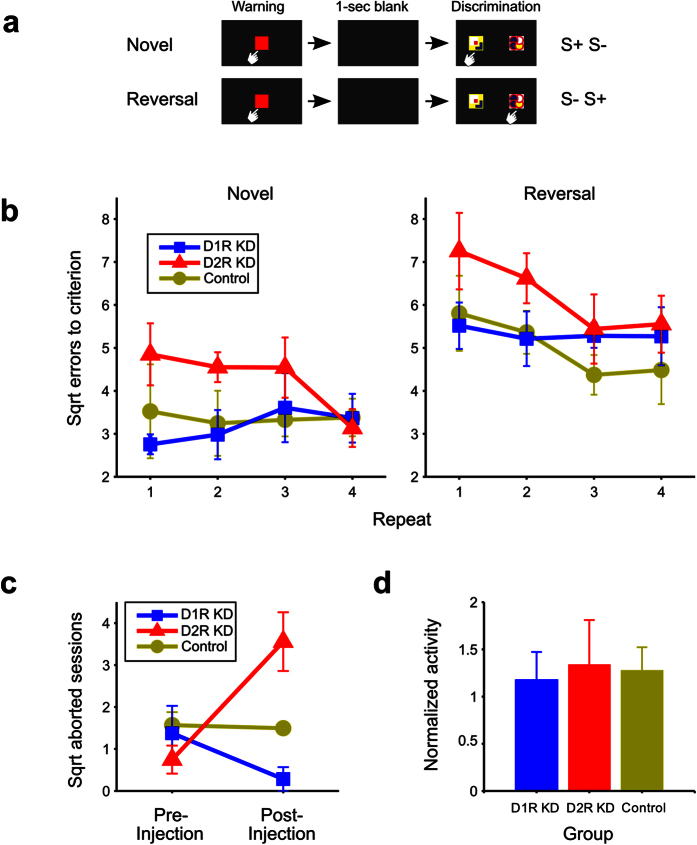
Behavioral assessments. (**a**) Schematic representation of a trial in a visual discrimination and reversal learning task as described in Methods section. (**b**) Square root errors (mean ± SEM) to criterion was calculated as the square root of the number of the errors until the subject reach to 90% correct responses in each session of trials of novel visual discrimination and reversal learning for D1R KD, D2R KD, and control marmosets (see Methods). (**c**) Mean number of aborted sessions (square root ± SEM) in pre- and post-injection periods. (**d**) Daily locomotor activity during the first 3 days of the post-injection period normalized to those during the last 3 days of the pre-injection period (mean ± SEM).

To directly determine the relationship between the caudate nucleus and cognitive function, as previously proposed and pharmacologically shown^3^,^14^,^15^,^16^, we compared the task performances before and after knocking down caudate D1R or D2R mRNAs. In the visual discrimination learning task, the marmosets were required to learn which of two visual patterns was associated with a reward. The task events in a trial are illustrated in Fig. 3a. In reversal learning, the stimulus–reward association was opposite that in the preceding learning. We conducted the novel discrimination learning task twice (N1 and N2) and then four repeats of the novel and reversal learning tasks (N3:R3 to N6:R6) before shRNA injections. As mentioned above, PET images of the marmoset brain with D1R- and D2R- specific ligands were captured before (pre-injection) and 1 month after (post-injection) shRNA injections (Supplementary Fig. 2c). Each shRNA injection resulted in a site-specific and receptor subtype-selective reduction in BPnd (Figs 1 and 2). Each marmoset was then subjected to four additional novel discrimination (N7 to N10) and reversal learning (R7 to R10) tasks after the injections.

After the injection, D2R KD resulted in significant impairments in both novel visual discrimination and reversal learning. As shown in Fig. 3b, the D2R KD marmoset group committed more errors to criterion than the D1R KD or control groups. A three-way analysis of variance (ANOVA) revealed significant main effects for GROUP (D1R-KD, D2R-KD, and control, *F*_(2,11)_ = 4.597, *P* = 0.035) and TASK (novel and reversal learning, *F*_(1, 11)_ = 50.192, *P* < 0.0001), but not for REPEAT (*F*_(3,33)_ = 1.271, *P* = 0.300). There were no significant interactions among these factors. Post hoc comparisons showed that D2R KD marmosets committed significantly more errors than D1R KD (*P* = 0.025) and control marmosets (*P* = 0.025), whereas there was no significant difference in the number of errors committed by D1R KD and control marmosets (*P* = 0.883).

In contrast to their behavior before the injection, D2R KD marmosets did not approach the testing apparatus for a long time (more than 15 min). To quantify this behavioral change, we counted the number of aborted sessions (see Methods) for each marmoset. The average number of aborted sessions before and after the shRNA injections for each group is shown in Fig. 3c. The number of aborted sessions significantly increased after the shRNA injections in the D2R KD marmosets [t(4) = 2.842, *P* = 0.047], whereas there was no significant change in the number after the injection for the D1R KD marmosets [t(4) = 1.305, *P* = 0.262], or control marmosets [t(3) = 0.209, *P* = 0.848]. After the shRNA injection, the number of aborted sessions significantly differed among the three groups [*F*_(2, 11)_ = 13.147, *P* = 0.001], and post hoc comparisons showed that the D2R KD marmosets aborted more sessions than the D1R KD (*P* < 0.001) and control marmosets (*P* = 0.012), whereas there was no significant difference between D1R KD and control marmosets (*P* = 0.104).

We further analyzed timing of abortion (the trial number) of D2R KD marmosets. Although four out of five marmosets aborted in the former half of sessions, the variances were so large and there was no specific tendency (Supplementary Fig. 4). For example, M7 ceased earlier trials but M8 stopped any time of trials.

It is unlikely that these specific effects of D2R KD were due to motor deficits because the locomotor activity of the D2R KD marmosets did not decrease after the shRNA treatment (Fig. 3d, one-way ANOVA, *F*_(2, 11)_ = 0.050, *P* = 0.952). In addition, the D2R KD marmosets revealed a strong appetite for the reward both before and after the receptor knockdown, and thus appeared not to lose their interest in the reward.

**Figure 4:**
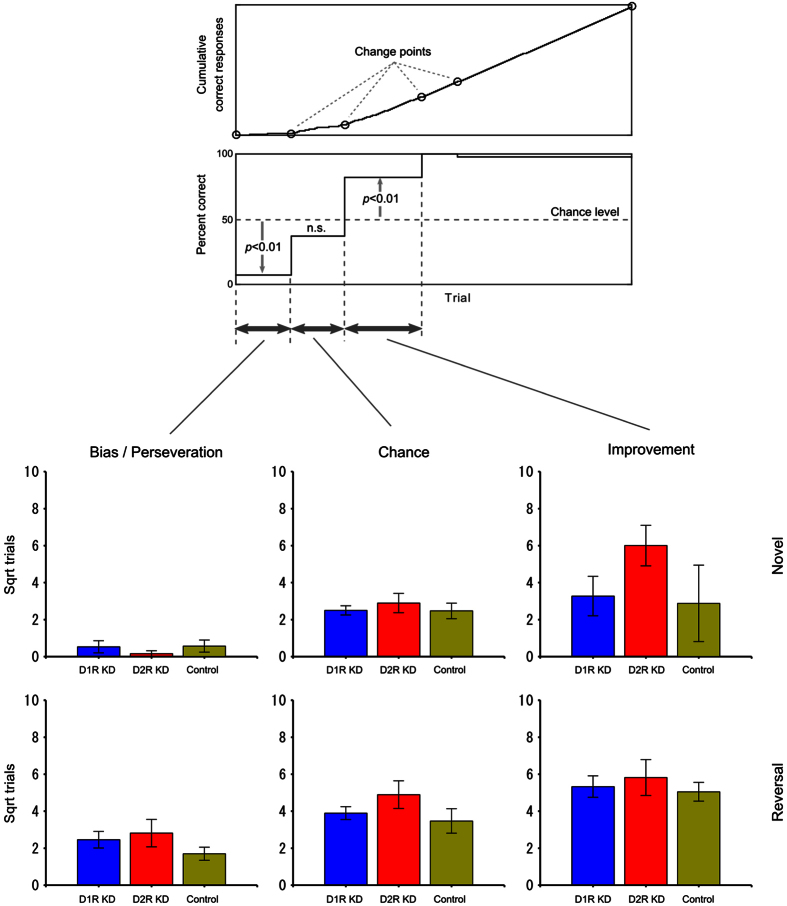
Comparison of the number of trials at various stages of learning. Top panels: The three stages were identified using the method of Takemoto *et al.*^66^. The “bias/perseveration” stage was also defined in the present study as the interval from the first trial to the last trial in which the correct ratio was significantly below the level of chance (α = 0.01). Bottom panels: The mean square roots of the number of trials at the three learning stages are plotted against the experimental groups. The blue, red, and yellow bars represent the results from D1R KD, D2R KD, and control marmosets, respectively. The upper and lower rows show the results of novel visual discrimination and reversal learning, respectively. The error bars represent SEM (±). For all learning stages in novel and reversal learning, the square root of the number of trials among the three groups was compared using one-way ANOVA. As shown in the Table 3 and Fig. 3 of original paper^66^, the wild type marmosets could learn in very early onset trails when once they start to learn.

To examine which stages in learning were impaired for D2R KD marmosets, the number of trials was separately examined at three learning stages: “bias/perseveration”, “chance”, and “improvement” (see Methods). In Fig. 4, the mean square roots for the number of trials at the three learning stages are plotted for the experimental groups. The average number of trials for D2R KD marmosets is highest at all learning stages, except for the “bias” stage in novel learning. Indeed, we found some higher errors in the improvement phase of N7, N8 and N9 and the perseveration of R7. However, no statistically significant group difference was detected (α = 0.05) (Supplementary Fig. 5).

**Figure 5:**
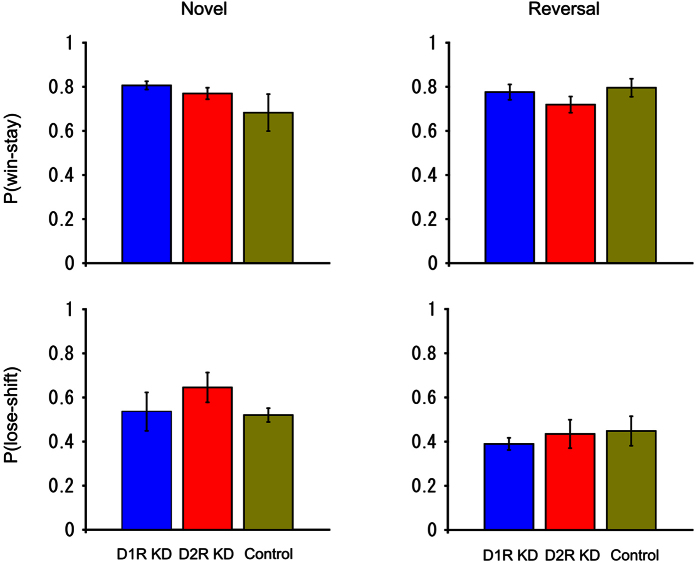
Win-stay/lose-shift analysis. The upper panels represent the win-stay probabilities and the lower panels show the lose-shift probabilities. The left and right panels display the results in the novel and reversal learning, respectively. The blue, red, and yellow bars represent the results from D1R KD, D2R KD, and control marmosets, respectively. For each marmoset, the probabilities were calculated using the trial data at all three learning stages (described in Fig. 4) in the four repeats for novel and reversal learning. The probabilities were then averaged across animals. The error bars represent SEM (±). The win-stay and lose-shift probabilities among the three groups were compared using one-way ANOVA.

To further investigate the possibility that stimulus stickiness increased in D2R KD marmosets, the win-stay and lose-shift probabilities were analyzed (Fig. 5). The win-stay probability is the probability that the animal touched the same stimulus in the next trial as that of the rewarded stimulus in the immediately preceding trial. The lose-shift probability is the probability that, after the animal was not rewarded by touching one stimulus, the animal touched the other stimulus in the next trial. No significant group difference was found in the win-stay or lose-shift rates for novel or reversal learning (one-way ANOVA, *F*_(2, 11)_ = 1.836, *P* = 0.205 for win-stay in novel learning, *F*_(2, 11)_ = 1.147, *P* = 0.354 for win-stay in reversal learning, *F*_(2, 11)_ = 0.957, *P* = 0.414 for lose-shift in novel learning, *F*_(2, 11)_ = 0.317, *P* = 0.735 for lose-shift in reversal learning).

**Figure 6:**
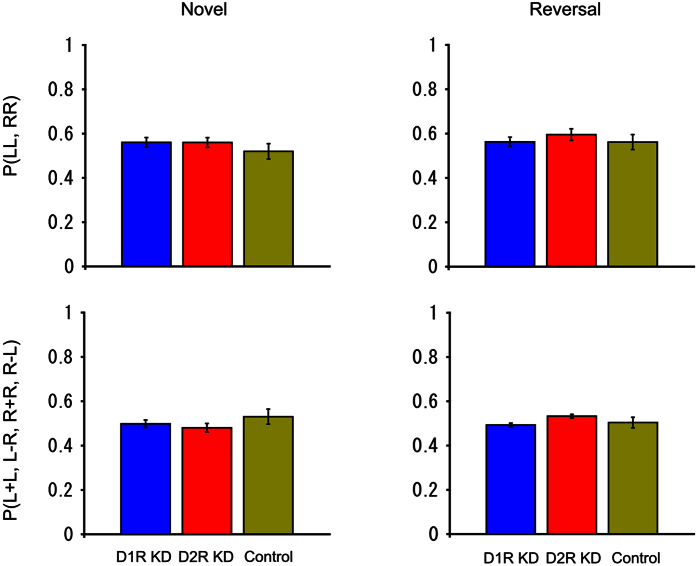
Comparison of side stickiness. The simple side-stickiness probabilities are plotted in the upper panels and the reward-associated side-stickiness probabilities are plotted in the lower panels. The left and right panels show the results in novel and reversal learning, respectively. The blue, red, and yellow bars represent the results from D1R KD, D2R KD, and control marmosets, respectively. The error bars represent SEM (±). The trial data used in this analysis were the same as those used in Fig. 5. The probabilities of the three groups were compared using one-way ANOVA.

To examine the effect of D1R and D2R knockdown on the side stickiness of responses, two probabilities were calculated. One is the simple side-stickiness probability that the marmoset touched the same side (left or right) in two consecutive trials. The other is the reward-associated side-stickiness probability, that is, the animal was rewarded if the same side that was touched in the trial immediately preceding it was again touched, but was not rewarded if the other side was touched in the next trial. No significant group difference was found for either probability in novel or reversal learning (one-way ANOVA, *F*_(2, 11)_ = 0.969, *P* = 0.410 for simple side-stickiness in novel learning, *F*_(2, 11)_ = 0.536, *P* = 0.600 for simple side-stickiness in reversal learning, *F*_(2, 11)_ = 0.202, *P* = 0.820 for reward-associated side-stickiness in novel learning, *F*_(2, 11)_ = 2.009, *P* = 0.180 for reward-associated side-stickiness in reversal learning) (Fig. 6). This result indicates that it would be difficult to attribute the learning deficits of D2R KD marmosets to side stickiness.

### Alteration of response time and time shift of activity in D2R KD marmosets

**Figure 7:**
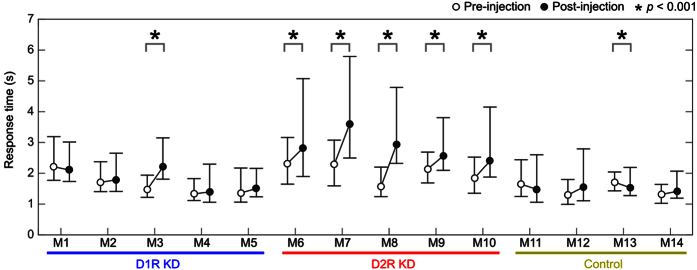
Comparison of touch response times from the presentation of two visual stimuli between before (N3-R6) and after (N7-R10) AAV2-shRNA injection. The response times in the last twenty trials in each experiment were pooled and compared using Mann-Whitney’s U-test. Open and filled circles represent the median value of the response time before (N3-R6) and after (N7-R10) AAV2-shRNA injection, respectively. Error bars show the first and third quartiles. Control, control marmosets; D1R KD, dopamine D1 receptor knockdown marmosets; D2R KD, dopamine D2 receptor knockdown marmosets.

We have examined other phenotypic alterations of D2R KD marmosets. First, we investigated response time among D1R KD, D2R KD and control marmosets. We show the performance and response time of each of all the examined marmosets (Supplementary Fig. 6). Compared with control and D1R KD marmosets, D2R KD marmosets seem to have shown a tendency of longer response times even when once their response times reduced. We therefore compared the response time of final 20 trials of four repeats (N7 to N10 and R7 to R10 in total 160 trials) (Fig. 7). All the post DR2 KD marmosets (5/5) showed statistically significant slower response time than pre D2R KD marmosets (Mann–Whitney U test: *P* < 0.001). Only one (1/5) D1R KD marmoset showed significant difference and one control marmoset showed statistically significant but decreased response time. These results indicate that post D2R KD marmosets significantly showed longer response time than D1R and control marmosets.

Secondly, we have analyzed the distributed pattern of daily locomotor activity of D1R KD, D2R KD and control marmosets (Supplementary Fig. 7a). We calculated the weighted center of the total amount of the activity per day (24 hr). The weighted center (red line) of post D2R KD marmosets shifted later hours than that of pre-injected marmosets. The statistical analysis (Mann–Whitney U test) showed significant shift to later time four out of five D2R KD marmosets (Supplementary Fig. 7b). In contrast, two Post D1R KD marmosets showed statistically significant (*P* < 0.001) but to both directions: one was to later, but the other was to earlier. One control marmoset showed statistically significant alteration (*P* < 0.001) but to the opposite direction (earlier than pre-injection one).

We further analyzed the nighttime activity for three hours after the onset of dark (Supplementary Fig. 7c). This figure indicates that D2R KD marmosets had a tendency (4/5) to more activity in dark compared to D1R KD and control marmosets. These data indicate that post-injected D2R KD marmosets became more active in the later time of the day compared to the post D1R KD and control marmosets.

### D2R BPnd is associated with learning performance

**Figure 8:**
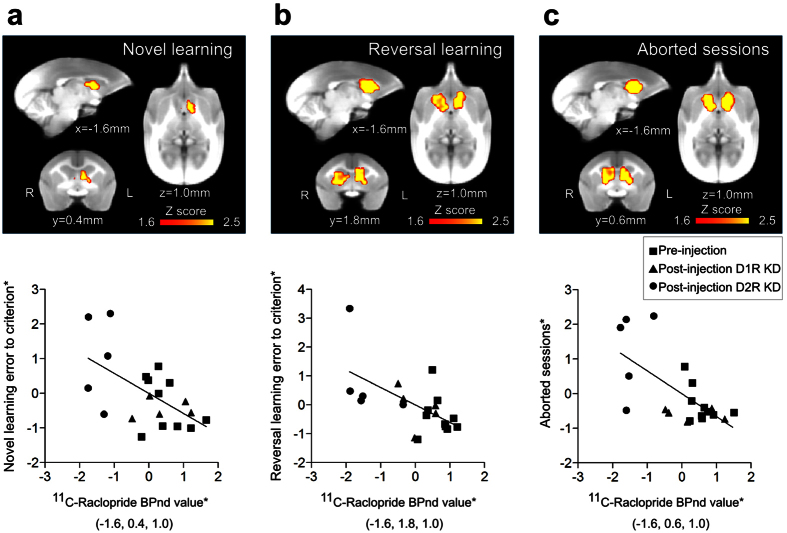
Voxel-based statistical results for the association of BPnd with behavior variables. Top panels: Yellow-red clusters indicate areas in which ^11^C-raclopride binding is correlated with novel visual discrimination learning errors to criterion (**a**), reversal learning errors to criterion (**b**), and aborted sessions (**c**). Bottom panels: Scatter plots show the correlation between ^11^C-raclopride binding within the caudate nucleus and behavioral variables. Clusters showing associations of BPnds with reversal learning and aborted sessions were identified in the rostral caudate nucleus coinciding with the injection sites (**b**,**c**). Regression relation was shown by a linear least square method in each behavioral variable; t(18) = −3.00, *P* < 0.0077 (**a**), t(18) = −3.22, *P* < 0.0048 (**b**), t(18) = −3.72, *P* < 0.0015 (**c**). The values of vertical and horizontal axes (*) represent so that data points were residualized and standardized individual values for pre- and post-injection of the peak voxels at the coordinates shown in parentheses.

To evaluate the correlation between the reductions in D1R and D2R availability and behavioral changes, we performed a voxel-based analysis of PET BPnd images, with behavioral variables as regressors (see Methods). Statistical analysis using all PET data from before and after shRNA administration indicated that D2R binding in the rostral caudate nucleus, which substantially matched the shRNA injection sites, was associated with novel visual discrimination and reversal learning performances and number of aborted sessions (*P* < 0.05, uncorrected; Fig. 8 and Supplementary Table 1), although the peak cluster associated with novel learning was located caudal to the shRNA injection sites (Fig. 8 and Supplementary Table 1). By contrast, neither novel nor reversal learning performance showed any association with D1R binding.

## Discussion

In this study, we applied the shRNA-mediated RNA interference technique to the regional KD of a target RNA in marmosets used as a primate model. We chose the caudate nucleus and the dopamine receptors D1R and D2R as the target region and molecules, respectively, because D1R and D2R play major roles in the caudate, which is separated from the putamen in primates. We chose AAV2 capsids to deliver shRNAs driven by the U6 promoter in this study, because among AAV serotypes AAV2 is less toxic than other serotypes when the CMV promoter is used^23^. We suppose that AAV2 causes less neuronal degeneration, although we did not conduct a comprehensive analysis compared with other serotypes in the marmoset striatum.

ISH results clearly demonstrated the efficient KD of D1R and D2R mRNAs in a wide region of the caudate bilaterally. Although an exact quantitative evaluation by ISH is difficult without using autoradiograms, which we did not use, it appears that PET results showed somewhat less reduction of D1R and D2R bindings than ISH results. D1R or D2R BPnd in the non-injected site (putamen) also decreased, which was more prominent in D1R KD marmosets (Fig. 1b). Since our ISH analysis did not indicate the reduced expression levels of D1R and D2R mRNAs in the non-injected site (Fig. 1a and Supplementary Fig. 3), there should be some mechanisms that underlie this discrepancy between PET and ISH results. Although ^11^C-SCH-23390 and ^11^C-raclopride are the most suitable PET ligands with excellent selectivity to D1R and D2R, respectively, in clinical PET^24^, equivalent binding affinities to D1R and D5R for ^11^C-SCH-23390 and to D2R and D3R for ^11^C-raclopride were also reported as *in vitro* pharmacological properties. Since D5R mRNA is expressed in the striatum with the highest level found in the monkey brain^25^, not only D1R but also D5R may contribute to the BPnd of ^11^C-SCH-23390 observed *in vivo* PET imaging. Another possible mechanism is that the decrease in the expression levels of caudate D1R and D2R mRNAs, particularly in that of D1R mRNA, could affect the dopamine neurotransmission in the putamen (non-injected site) via cortico-striatal-thalamic neural circuits. This might enhance dopamine release in the putamen leading to the BPnd reduction by receptor internalization^26^ or by competitive binding with PET ligands. For example, it is reported that orbitofrontal dopamine depletion causes tonic upregulation of caudate dopamine release^16^. Recent multimodal analysis of the striatum-substantia nigra (SNc) connectivity in mice^10^ revealed the connections of SNc with DMS and DLS, and identified two different subpopulations of parallel nigrostriatal dopaminergic neurons based on biophysical properties and both input and output wirings to DMS and DLS. This study shows that DLS corresponding to the putamen in mice and DMS corresponding to the caudate in mice preferentially projected to the corresponding SNc regions. However, there is also intermingling of the projections between DLS, SNc, and DMS. Therefore, it is possible that the decrease in the caudate D1R mRNA expression level affects the putamen D1R BPnds (Fig. 1b).

We observed the altered behaviors of D2R KD marmosets but no apparent phenotype alteration in D1R KD marmosets. The altered phenotypes in D2R KD marmosets were wider than previously reported^16^,^27^. The performance and the response time of each of marmosets examined, the response time of D2R KD seems to have been more fluctuated than those of D1R KD and control marmosets (Supplementary Fig. 6). We found that the response time of the last 20 trials of each session of all post-injected D2R KD marmosets (5/5) was significantly slower than that of pre-injected marmosets (Fig. 7). The numbers of the aborted session that was defined if a marmoset did not touch the screen for 15 min increased. We examined when this aborted sessions occurred during the trials and found this could happen at various timing (Supplementary Fig. 4). We did not find any indication of impairments of locomotion and appetite for rewards in D2R KD marmosets after (post) injection. Therefore, these observations implicate that the caudate D2R KD caused an erratic response rather than impairing motivation and locomotion. This erratic response to the tasks may be consistent with the fluctuating response time observed (Supplementary Fig. 6). It has been proposed that dopamine release in the dorsal striatum sets clock speed or internal timing^28^. The timing may be controlled in the striatum both action initiation and cognitive decision-making^29^. Therefore these data raise an intriguing possibility that D2R in the caudate plays a critical role in the cognitive initiation, and the D2R impairment disturbs the timing of initiation by reducing tonic activity.

The mechanisms why the daytime activity shifted to the nighttime activity also need to be examined. Sleep disturbances is reported in Parkinsonisms^30^. The relevance of the shift of daytime activity towards nighttime in D2R to the sleep disorder with Parkinsonism remains to be studied. D1R KD marmosets did not show any apparent deficits in discrimination, reversal learning, or any changes in aborted attempts in these tasks.

Several lines of evidence from previous studies suggest a correlation between caudate D2R and its cognitive function^16^,^27^,^31^,^32^. First, Pessiglione *et al.*^33^ demonstrated that the administration of 3,4-dihydroxy-L-phenylalanine (L-DOPA) and haloperidol respectively enhances and attenuates dopaminergic function. Consequently, enhancing dopaminergic activity improves choice performance towards monetary gains, but not avoidance on monetary loss^33^. Lee *et al.*^34^ found that D2/D3 receptors play a specific role in the reversal of a learned visual discrimination in monkeys^34^. Jocham *et al.*^35^ found reversal-learning deficits in human carriers of the A1 allele of TaqIA polymorphism in the D2R genes^35^. Frank and O’Reilly^36^ reported that D2-like receptor agonists affect behavioral adjustment, presumably by impairing Go learning from positive reinforcement, and D2-like antagonist in the opposite direction^36^. Moreover, Frank and Hutchison^37^ demonstrated that human TaqlA A1 allele carriers show impaired responses to negative feedback, which suggests that low D2-like receptor response levels may give rise to inflexibility due to alteration of feedback sensitivity^37^. Second, Groman *et al.*^31^ used three choices of boxes with the lid being associated with visual cues^31^. The outcome of tasks was either a correct choice, an incorrect choice, or an omission. PET analysis revealed that D2-like receptor availability in the dorsal striatum was not related to individual differences in the ability to acquire or retain visual discrimination, but was related to the number of trials required to reach the criterion in the reversal phase of the task. D2-like receptor availability also strongly correlated with behavioral sensitivity to positive feedback, but not to negative feedback, during learning^31^. Our results are consistent with those of Groman *et al.*, because both forward and reversal learning are impaired (Note that both forward and reversal learning can be affected by positive feedback but not by negative feedback in relation to D2R availability^31^). Their results demonstrated the correlation, and not the dissociation of receptor availability from dopamine concentration. On the other hand, our D1R KD and D2R KD systems directly demonstrated that the postsynaptic decrease in the expression level of caudate D2R mRNA affected both forward and reversal learning. Third, Eisenegger *et al.*^32^ further revealed the role of D2R, that is, it selectively affects stimuli related to a positive reward but not to a negative reward at a high dose of sulpiride (a D2R/D3R antagonist), which sufficiently provides the postsynaptic occupancy of D2R, in combination with human D2R genotype alleles^32^. For their analysis, they used a Q-learning algorithm with the subjective values of Q_t_^A^ and Q_t_^B^, which are the expected rewards for choosing between stimuli A and B, respectively, updated with the feedback that subjects receive (R_i-1_) in each trial, with the formulation Q_t_^A^ = (1 − α)Q_1-i_^A^ + αR_t-1_. The softmax function for reinforcement learning^38^ was then used to estimate the probability to choose either stimulus, and maximum likelihood methods were used to estimate the learning rate (**α**) and temperature (β). Their results showed that sulpiride did not affect α (learning rate), but selectively increased β (temperature), which suggests impairments in choice performance. Our results are consistent with those of Eisenegger *et al.*^32^, although the task and the method they used were different from ours. We used a pair of stimuli to elicit a correct choice for our tasks, while they used monetary tasks that consisted of two domains of gain and loss, in which each of the two pairs of stimuli was associated with one of the pairs of outcomes. Concern about the specificity of the impairment of D2R, because of the fact that sulpiride can bind to both D2R and D3R, was further confirmed by Eisenegger *et al.*, who showed that the only enhanced effect was the link to the A1 allele + subgroup of D2R Taq1A polymorphism with sulpiride administration. They found no effect of sulpiride on the loss domain of the monetary task. These findings led to the conclusion that there is no alteration in the learning parameter α, which posed difficulty in understanding the role of D1R using the currently prevailing model, which predicts a major role of D1R and supports studies of Parkinson’s disease patients^39^. Finally, Lee *et al.*^27^ reported the injection of either a D1R antagonist (SCH23390) or a D2R antagonist (eticlopride) into the dorsal striatum in macaque monkeys and the subsequent behavioral changes^27^. They found that the D2R antagonist affected choices based on previous outcomes (for fixed patterns), but not choices driven by perceptual inference (for random patterns). Their report supports the possibility of a specific role of striatal D2R in learning from past reinforcement, but a limited role in the perceptual inference task. As for evidence supporting the role of D1R, Lee *et al.*^27^ found no effect of a D1R antagonist on perceptual inference or reinforcement learning^27^. Our results further extend the evidence for the role of D1R beyond the results reported by Lee *et al.*, D1R KD in marmosets affects neither forward nor reversal learning, which excludes the possibility that the tasks were driven only by a negative outcome, contrary to the discussion by Lee *et al.*^27^, but supports the possibility that positive feedback learning was not affected in our tasks examined in D1R KD marmosets, which showed no significant difference in square root errors to reach the criterion from control marmosets, whereas D2R KD marmosets showed significant impairments in both novel and reversal learning (Fig. 3). In Supplementary Fig. 6, none of the D1R KD marmosets showed any significant differences in percent correct responses and reaction time as a function of trial number compared with the control marmosets, whereas all D2R KD marmosets showed quite variable responses as a function of trial number. Eisenegger *et al.*^32^ discussed the roles of D1R to explain why α (learning rate) does not change following the administration of sulpiride, which affects D2-like receptors^32^. They suggest the possibility that D1R, which is activated by phasic activity, has a more specific role than D2R because it has a relatively low binding affinity for DA and is more closely related to phasic DA release following unexpected rewards^40^. On the other hand, D2R has high affinity for DA binding^41^ and a closer relationship with the tonic activity of dopamine. This tonic activity may be linked to vigor and motivation^42^. They consider that D2R impairment does not affect acquisition of learning but does affect the expression of the learned behavior in the form of impaired choice performance and increased response time. Although they did not verify these predictions by themselves, the results of our present study confirmed these predictions, which may also add evidence to the relative roles of dopamine in rewards and punishment in multiple pathways in primates and rodents^10^,^43^,^44^,^45^,^46^,^47^. The phenotypes of D1R KD marmosets were in distinguishable from those of wild-type marmosets, whereas the phenotypes of D2R KD marmosets showed various differences from those of wild-type marmosets, which suggests that D1R plays a specific role in the caudate compared with D2R.

Despite an increasing number of studies using genetic manipulation methods in mice, there have been only a limited number of studies that directly manipulate either D1R or D2R in DMS (corresponding to the caudate in mouse). This is because no regional specific marker of DMS is established in rodents. Conventional D1R and D2R knock out (KO) mice show opposite phenotypes in motor and cognitive tasks. In motor tasks, D1R KO mice generally show more severe impairment than D2R KO mice^48^, whereas D2R KO mice show more severe impairment in choice behavior in an instrumental learning task with progressively increasing reversal frequency in a dynamic two-armed bandit task, than D1R KO mice^49^. Although the caudate D1R KD marmosets did not show any impairment, the conventional D1R KO mice showed some impairment in cognitive learning (choice behavior in an instrumental learning task). However, note that there is no study of the regional DMS KO of D1R and D2R, because no DMS-specific driver mouse line is as yet available, to the best of our knowledge. The ablation of D1R-positive DMS neurons impairs exploration of novel objects but not that of familiar objects and object memory^9^. Optogenetic stimulation of D1R-expressing medium spiny neurons (MSNs) induces persistent reinforcement, whereas stimulation of D2R-expressing MSNs induces transient punishment^50^. Therefore, there are some discrepancies in the cognitive phenotype between the receptor level (D1R KD) and the neuron level of ablation, and in the activation of the D1R-mediated direct pathway related to the presumed caudate (DMS) function. The D1R KD marmosets show no alteration of phenotypes, whereas direct activation and inactivation of MSNs show significant alteration of phenotypes. There may be several possible explanations for this discrepancy. First, the cognitive tasks used are different among the papers reported. Therefore, one can argue that the negative feedback task is not affected by D1R impairment as Lee *et al.*^27^ suggested^27^. However, this unlikely explains why there is no alteration of phenotypes of a wider range in D1R KD marmosets, as already discussed above. Second, species differences might be a possible explanation. Although we think that this explanation is possible, it is rather unlikely given that there is essential similarity between primates and rodents in striatal function. Third, there is another type of D1-like receptor (D5R) in the macaque caudate^25^,^51^, which may compensate for the activation of the direct pathway in the striatum. Note that once MSNs of the direct or indirect pathway are optogenetically activated, even the application of both D1R and D2R antagonists does not induce any interference^50^. Fourth, the residual D1R expression that escaped shRNA interference may contribute to the maintenance of the function of D1R. Judging from the ISH results (Supplementary Fig. 3), a similar level of caudate D2R mRNA KD causes significant behavioral changes. Therefore, the threshold of a decreased receptor mRNA expression level may be different between D1R and D2R. The absence of phenotype changes of D1R KD marmosets suggests more specific roles of caudate D1R in certain tasks than of D2R, as pointed out by Eisenegger *et al.*^32^. Other than our study using shRNA KD marmosets to directly examine the behavioral outcome of D1R impairment, there has been only one report of experiments by Lee *et al.*^27^ using antagonists, and therefore it is impossible to compare our results with other results obtained using different tasks at this point. However, if a wider variety of tasks are examined using D1R KD marmosets, we expect that it will become clear as to what kind of tasks whose performance is impaired by D1R KD.

In conclusion, the results of caudate D1R KD and D2R KD taken together extend our understanding of the role of D2R and shed new light on the functions of D1R, which may not be simply explained using currently prevailing models of the striatum^39^. It therefore raises the question on the underlying mechanisms of caudate D1R functions, which remains to be clarified in future studies. These studies should include well-designed tasks based on theoretical predictions by multidisciplinary approaches that include anatomy, electrophysiology, optogenetics, and two-photon imaging in combination with our shRNA-mediated KD systems.

## Methods

### Animals

Fourteen naive common marmosets (*Callithrix jacchus*, five males and nine females, aged between 2 and 3 years) were used in this study. Each marmoset was housed in an individual cage placed in an isolator rack system (Natsume Seisakusho Co., Ltd., Tokyo, Japan) at the Primate Research Institute, Kyoto University. The light was on from 7:00 until 19:00. Behavioral experiments were conducted between 13:00 and 16:00 on weekdays. The animals were fed 30 g of New World monkey pellets once daily. On weekends, they were also fed supplements (banana, apple, raisin, steamed sweet potato, egg, mealworms, and gum with VD). Water was provided *ad libitum*. All procedures were conducted in accordance with the guidelines for laboratory animals by the National Institutes of Health and the Ministry of Education, Culture, Sports, Science and Technology (MEXT) of Japan, and the *Guide for Care and Use of Laboratory Primates* by the Primate Research Institute, Kyoto University. The marmoset experiments were also approved by the Institutional Animal Care and Use Committee of the National Institutes of Natural Sciences, the local Animal Experimental Committee of Kobe Institute of RIKEN, and the Ethics Committee of the Primate Research Institute, Kyoto University.

### Construction of plasmid vectors

AAV vectors were based on the AAV helper-free system (Agilent Technologies, CA, USA). To generate plasmids encoding shRNA driven by a U6 promoter, a 0.7-kb *Pvu*II fragment from pSilencer 2.1-U6 NEO (Ambion Life Technologies, MA, USA) was inserted into the blunted *Mlu*I site of pAAV-hrGFP (Agilent Technologies)^52^. The CMV promoter for hrGFP was replaced by the human synapsin I promoter. A shRNA cassette was inserted into the plasmid digested with *Bam*HI and *Hind*III located directly below the U6 promoter^52^. The shRNA target sequence for the marmoset D1R was 5′-GTCGAATGTTCTCAACCAGAA-3′, and that for the marmoset D2R was 5′-GGACAGACCTCACTACAATTA-3′. Silencing efficacy was evaluated using the Dual-Luciferase reporter assay system (Promega, WI, USA)^53^. The shRNAs with more than 80% knockdown efficacy *in vitro* were identified (see Supplementary Fig. 1), and those having high efficacy were tested more than twice.

### Preparation and injection of AAV into marmoset caudate nucleus

The AAV-2 were prepared as described previously^54^. They were produced in HEK293 cells using a helper-virus-free system, and purified twice with CsCl_2_ density gradients and titrated by Q-polymerase chain reaction (PCR). Final preparations were dialyzed against 50 mM HEPES-buffered saline. To prevent adhesion of the AAV vector to the glass micropipettes, 0.001% Pluronic-F68 solution (Sigma-Aldrich, MI, USA) was added to the vector solution. Basic techniques for AAV injection were followed as previously described^55^. Anesthesia was induced using intramuscular injections of ketamine hydrochloride (Ketalar, 30 mg/kg, Daiichi Sankyo, Tokyo, Japan) and medetomidine hydrochloride (Domitor, 0.06 mg/kg, Orion Pharma, Berkshire, UK), followed by premedication with carprofen (Rimadyl, 4.4 mg/kg, Zoetis, NJ, USA), carbazochrome sodium sulfonate hydrate (Adona, 0.2 mg/kg, Mitsubishi Tanabe Pharma, Osaka, Japan), ampicillin sodium (Viccillin, 34 mg/kg, Meiji Seika Pharma, Tokyo, Japan), and 10 ml of lactated Ringer’s solution (Solulact, Terumo,, Tokyo, Japan) containing 0.4 mg of riboflavin sodium phosphate (Bisulase, Fuji Pharma, Tokyo, Japan). Under deep anesthesia induced by isoflurane (1–2%) inhalation (Wako, Osaka, Japan), the head of the marmoset was fixed to the stereotaxic apparatus and the operation was performed. Heart rate, O_2_ saturation, and body temperature were continuously monitored. A glass micropipette (tip diameter, 100 μm) was filled with the AAV vector solution. The injections were aimed at the medial anterior caudate nucleus in both hemispheres. The AAV vector solution (4 × 10^9 ^vg/μl, 2μl each) was injected over 10 min, and this was controlled using a syringe pump. After termination of the injection, the pipette was fixed at the same position for another 2 min and then pulled out. Four injections in two tracks in each hemisphere were made into the medial anterior caudate nucleus at 1 mm intervals. After the injections, the dura was covered with Spongel (Astellas, Tokyo, Japan), and the skin was sutured closed. After the operation, gentamicin sulfate (Merck Sharp and Dohme, NJ, USA) and enrofloxacin (Baytril, 15 mg/kg, Bayer HealthCare, Leverkusen, Germany) were administered. At the end of the experiments, the marmosets were deeply anesthetized with an intramuscular injection of ketamine, followed by an intraperitoneal injection of sodium pentobarbital. They were then transcardially perfused with saline followed by 4% paraformaldehyde in 0.1 M phosphate buffer (pH 7.4). The whole brain was cryoprotected and sectioned at a thickness of 40 μm using a freezing microtome.

### ISH

Coronal sections were cut into 40 μm-thick slices. Digoxigenin (DIG)-labeled riboprobes were produced using template plasmids, which included the PCR fragments generated using the following primers:

Marmoset D1R -1f: 5′-ATGAGGACTCTGAACACCTC-3′

Marmoset D1R -1r: 5′-GTGACAATCATGATGGCCAC-3′

Marmoset D1R -2f: 5′-TACACCAGGATCTACAGGAT-3′

Marmoset D1R -2r: 5′-AGCTTCTCCAAGGGTCTGGC-3′

Marmoset D2R -1f 5′-ATGGATCCACTGAATCTGTC-3′

Marmoset D2R -1r: 5′-ATGAAGGGCACGTAGAAGGA-3′

Marmoset D2R -2f: 5′-GTCACCCTGCTGGTCTACAT-3′

Marmoset D2R -2r: 5′-TAGCCCAGCCACGTGAAGGC-3′.

ISH was conducted as previously described^56^,^57^,^58^. Briefly, free-floating sections were treated with proteinase K (5 μg/ml) for 30 min at 37 °C, acetylated, and then incubated in a hybridization buffer (5 × SSC, 2% blocking regent [Roche Diagnostics, Basel, Switzerland], 50% formamide, 0.1% N-lauroylsarcosine, and 0.1% SDS) containing 0.5 μg/ml DIG-labeled riboprobes at 60 °C. The sections were sequentially treated in 2 × SSC, 50% formamide, 0.1% N-lauroylsarcosine for 20 min at 60 °C twice, 30 min at 37 °C in RNase buffer (10 mM Tris-HCl [pH 8.0], 1 mM ethylenediaminetetraacetic acid, and 500 mM NaCl) containing 20 μg/ml RNase A (Sigma-Aldrich), 20 min at 37 °C in 2 × SSC/0.1% N-lauroylsarcosine twice, and 20 min at 37 °C in 0.2 × SSC/0.1% N-lauroylsarcosine twice. The hybridization probe was detected with an alkaline phosphatase-conjugated anti-DIG antibody (Cat. No. 11093274910) using a DIG nucleic acid detection kit (Roche Diagnostics). For ISH, two probes were prepared for each gene. We confirmed that the two probes for each gene exhibited essentially the same hybridization signal patterns and that D1R and D2R mRNAs were expressed in distinct populations of neurons in the striatum. After these confirmations, the two probes were mixed together to intensify the signals.

### PET procedure

In total, 20 PET scans were conducted (ten individual animals twice each, for pre- and post-injections of AAV). A small-animal PET scanner was used (microPET Focus220; Siemens Medical Solutions, Knoxville, TN, USA), with a 220 mm bore arranged in rings of contiguous discrete detectors with 190 and 78 mm transaxial and axial fields of view, respectively. The spatial resolution at the center of the field of view was approximately 1.35 mm for full width at half-maximum (FWHM). Animals were anesthetized using 1–2% isoflurane inhalation. The tail vein was cannulated for the PET ligand injection, and the head was fixed on the bed with the stereotaxic apparatus. Transmission scanning was performed for 30 min with a ^68^Ge-^68^Ga pin source for attenuation correction of emission scans. A dynamic histogram was acquired during the 90 min (6 × 10 s, 6 × 30 s, 11 × 60 s, 15 × 180 s, and 3 × 600 s) immediately after the bolus injection of radioactive tracers. Images were acquired in a three-dimensional list mode and reconstructed using an algorithm of filtered back projection smoothed using a Hanning filter at a cutoff of 0.5. An attenuation correction, but not scatter fraction using blank and transmission images, was performed to obtain quantitative images. The radioactive tracers ^11^C-SCH23390 and ^11^C-raclopride were used as the ligands for D1R and D2R, respectively. The ^11^C-SCH23390 was prepared by N-^11^C-methylation of the corresponding precursor, (+)-SCH24518, with ^11^C-methyl triflate as described previously^59^. Its radiochemical purity was > 90%, and its specific radioactivity was 66.4 ± 20.8 GBq/μmol (mean ± s.d.). The injected radioactivity was 166.3 ± 1.92 MBq/kg. The ^11^C-raclopride was prepared by O-^11^C-methylation of the corresponding precursor, (S)-3,5-dichloro-2,6-dihydroxy-N-((l-ethyl-2-pyrrolidinyl)methyl) benzamide hydrobromide, with ^11^C-methyl triflate as described previously^60^. Its radiochemical purity was > 94%, and its specific radioactivity was 70.5 ± 19.4 GBq/μmol (mean ± s.d.). The injected radioactivity was 163.6 ± 20.3 MBq/kg.

### MRI

To obtain individual brain anatomy images, MRI (3T, Allegra, Siemens, Erlangen, Germany) of the brain was performed on all animals. The template of the brain MR image, which we had previously prepared^61^, was used for the normalization of individual PET images for voxel-based analysis.

### PET image processing

Reconstructed PET images were processed using the image analysis software PMOD, version 3.504 (PMOD Technologies, Zurich, Switzerland). To quantify ^11^C-SCH23390 and ^11^C-raclopride binding activities, the BPnd was calculated using Logan’s reference tissue model with fixed k2′^62^. The k2′ was estimated by parametric analysis with a simplified reference tissue model using the cerebellum as a reference region and the caudate nucleus and the putamen as signal-rich regions^63^.

### PET image analysis

Individual PET images were aligned on each MR image by rigid matching using PMOD’s Neuro Tool PNEURO. PET images of the brain were deskulled according to the structural information from individual MR images, and then registered to the standard space of the template MR image by affine transformation with 12 degrees of freedom using a cost function of a normalized correlation ratio with the FLIRT (FMRIB’s Linear Image Registration Tool) component of FSL (FMRIB’s Software Library, www.fmrib.ox.ac.uk/fsl). To evaluate the reduction in BPnd after the shRNA treatments, the average BPnd was calculated in bilateral regions of interest (2 mm^3^ each) for injection sites and non-injection sites. The percentage change was calculated by dividing a difference between post-injection and pre-injection BPnd values by the pre-injection value, and then compared using t-test for injection sites and non-injection sites separately. Normality of data distribution was verified by Shapiro-Wilk test.

### Voxel-based statistics with behavioral variables

For detecting regions in which dopamine receptor binding correlated with behavioral variables, voxel-based statistical analysis was performed using the graphical user interface tool FEAT component of FSL. Before the analysis, all the parametric BPnd images were processed with motion correction and smoothing using a 2.0 mm Gaussian kernel at FWHM. The statistical modeling was based on a general linear model using one explanatory variable of behavioral score with a global mean value of the brain in PET image as a covariate. Three categories, novel learning errors to criterion, reversal learning errors to criterion, and the number of aborted sessions, were used as behavioral variables. Statistical images were masked by the striatum before thresholding, including the caudate nucleus and putamen, by referring to a marmoset brain atlas^64^. Normality of data distribution was verified by Shapiro-Wilk test, and the values in 99.8% and 97.4% voxels analyzed for D1R and D2R, respectively, were normally distributed. The *P*-value threshold was set at 0.05, uncorrected for multiple comparisons, and the resulting statistical images were registered to the template MRI image (Fig. 8 and Supplementary Table 1).

### Apparatus for visual discrimination learning

The presentation of stimuli, recording of responses, and delivery of rewards were all performed using a compact apparatus consisting of a 7 inch tablet computer and a USB-driven feeder^65^. The apparatus was attached to the cage in the isolator rack system during the experiment. The feeder delivered a small piece of a solid reward into a food tray below the screen. The marmosets reached their hands out of the cage to touch the screen of the tablet computer and then retrieved the reward from the food tray. Four types of rewards were used: marshmallow, sponge cake, fizzing candy, or cheese. One of these rewards was dispensed in every trial to keep the marmoset’s motivation level high. The behavior of the marmosets was monitored and recorded with a small video camera placed over the cage. In addition, to measure locomotor activity throughout an entire day, the Actiwatch Mini (CamNtech Ltd., Cambridge, UK), which recorded locomotor activity by means of an accelerometer, was attached to the marmoset’s neck using a collar. The activity data were sampled every 30 s.

### Behavioral training

All marmosets were trained to touch the stimulus on the screen rapidly and with a stable performance. Initially, a large colored square (7.5 cm) was presented at the center of the screen, which had a black background. The color of the square was randomly selected from red, blue, and yellow. When the marmoset touched the square, a tone (4 kHz, 100 ms) was emitted and a reward was given. After a 3 s intertrial interval in which the screen was blank, the next trial was started. A session consisted of 30 trials. A maximum of five sessions was performed in a day. Once a marmoset reliably touched the stimulus, the side length of the square was reduced by 1.5 cm, until the animals reliably touched a 3 cm square. Next, the marmoset was trained to touch the stimulus located at various spatial positions. This training continued until the time required for completing a session plateaued, which ranged from 3 to 5 min depending on the individual’s ability. Finally, to prevent the marmoset from hesitating to touch a novel image in visual discrimination learning, 30 different abstract visual patterns were used as stimuli instead of the uniformly colored square. The stimuli had the same colors and the same size as those used in visual discrimination learning but had different patterns. For this training, only one session was performed for each marmoset. After that, the Actiwatch Mini was attached to the marmoset, and a series of visual discrimination and reversal learning tasks were started.

### Visual discrimination and reversal learning

In novel visual discrimination learning, the marmoset was required to learn which visual pattern was associated with a reward. The task event in a trial is illustrated in Fig. 3a. At the beginning of a trial, a red square (2.7 × 2.7 cm^2^) appeared at the center of the screen against a black background as a warning signal until the marmoset touched it. Then, after a 1 s interval with a blank screen, a pair of square abstract graphic patterns in blue, yellow, red, and white were presented simultaneously to the left and right of the center of the screen^65^. The size of the patterns was 2.7 cm and the distance between the centers of the patterns was about 7.5 cm. The left–right positions were pseudorandomly changed and counterbalanced in a session. One of the patterns was always associated with a reward and the other was not. Once the marmoset touched either of the patterns, both disappeared. When the marmoset correctly touched the reward-associated pattern, a reward was delivered with a tone (100 ms, 4 kHz). The correct and incorrect responses were followed by 3 s and 5 s intertrial intervals, respectively. When the marmoset did not touch any stimulus for 15 min, the session was aborted and no more trials were conducted for the marmoset that day. A daily session consisted of 100 trials. When a criterion of 90% correct was attained, the marmoset was considered to have learned the first novel visual discrimination (N1). Thereafter, a new pair of visual patterns was introduced (N2). After the third novel visual discrimination (N3), its reversal problem (R3) was introduced. In reversal learning, the stimulus–reward association was opposite to that of the preceding novel learning. The same criterion for learning was adopted for the reversal task. This combination of the novel visual discrimination and reversal learning tasks was repeated four times (N3:R3 to N6:R6). Subsequently, the marmosets received AAV injections for D1R (*n* = 5) or D2R (*n* = 5) KD. Four nontreated marmosets were used as controls. After a period of recovery for the D1R- and D2R-KD marmosets or after a comparative interval for the control marmosets (more than 1 month), the Actiwatch Mini was attached again and the reversal task (R6) was retested. Then, the combination of the novel and reversal learning was repeated four times (N7:R7 to N10:R10). All 20 visual patterns as shown in the previous paper (Fig. 1B in Takemoto *et al.*^66^) were randomly assigned to ten discrimination tasks (N1 to N10) for each marmoset to minimize idiosyncratic effects.

To examine the effect of D1R or D2R KD, the square root of the number of errors to the criterion was calculated to eliminate variance heterogeneity (shown by Levene’s test). The values were then compared using three-way ANOVA, where the main factors were GROUP (D1R KD, D2R KD, and control), TASK (novel and reversal learning), and REPEAT (N7, N8, N9, and N10); GROUP was a between-subject factor and the others were within-subject factors. There was no significant sphericity violation (Mauchly’s test). Fisher’s LSD test was used for post hoc comparisons. For the comparison of the number of aborted sessions, the square-root transformation was used. The square root of the number of aborted trials between the pre-injection and post-injection was compared using a paired *t*-test. The three groups post-injection were compared using a one-way ANOVA with Fisher’s LSD test for post hoc comparisons. Considering the sensitivity to the Actiwatch Mini device among the marmosets, the same device was always used for a given animal. For comparison of locomotor activity among the three groups post-injection, the locomotor activity was normalized for each marmoset by dividing the daily activity during the first 3 weekdays post-injection by that during the last 3 weekdays pre-injection, because considerable individual differences in locomotor activity were observed among the marmosets. The group means of the normalized activity were then compared using one-way ANOVA.

We examined the number of trials at three learning stages: “bias/perseveration”, “chance”, and “improvement” using the method of Takemoto *et al.*^66^, in which a recursive algorithm was used to find change points in a cumulative correct response curve^67^. As illustrated in the upper part of Fig. 4, these three stages were identified. The bias/perseveration stage was also defined in the present study as the interval from the first trial to the last trial in which the correct ratio was significantly below the level of chance (α = 0.01). For each marmoset, the number of trials at each stage was calculated for each of the four repeats in the novel and reversal learning, and the four values were averaged, collapsing the effect of the repeats. Then, the three values for the novel and reversal learning were averaged across the animals after the square-root transformation to eliminate variance heterogeneity.

## Additional Information

**How to cite this article**: Takaji, M. *et al.* Distinct roles for primate caudate dopamine D1 and D2 receptors in visual discrimination learning revealed using shRNA knockdown. *Sci. Rep.* **6**, 35809; doi: 10.1038/srep35809 (2016).

**Publisher’s note**: Springer Nature remains neutral with regard to jurisdictional claims in published maps and institutional affiliations.

## Supplementary Material

Supplementary Information
