# Supplementary material for: Distinct roles for primate caudate dopamine D1 and D2 receptors in visual discrimination learning revealed using shRNA knockdown
Source: Sci Rep. 2016 Nov 2;6:35809. doi: 10.1038/srep35809 (PMC5090965; doi:10.1038/srep35809)
Supplement: Supplementary Information [file srep35809-s1.pdf]

Supplementary Information

Distinct roles for primate caudate dopamine D1 and D2 receptors in visual discrimination learning revealed using shRNA knockdown

Masafumi Takaji, Atsushi Takemoto, Chihiro Yokoyama, Akiya Watakabe, Hiroaki Mizukami, Keiya Ozawa, Hirotaka Onoe, Katsuki Nakamura, Tetsuo Yamamori

This file includes
- Supplementary Figures 1 - 7
- Supplementary Table 1

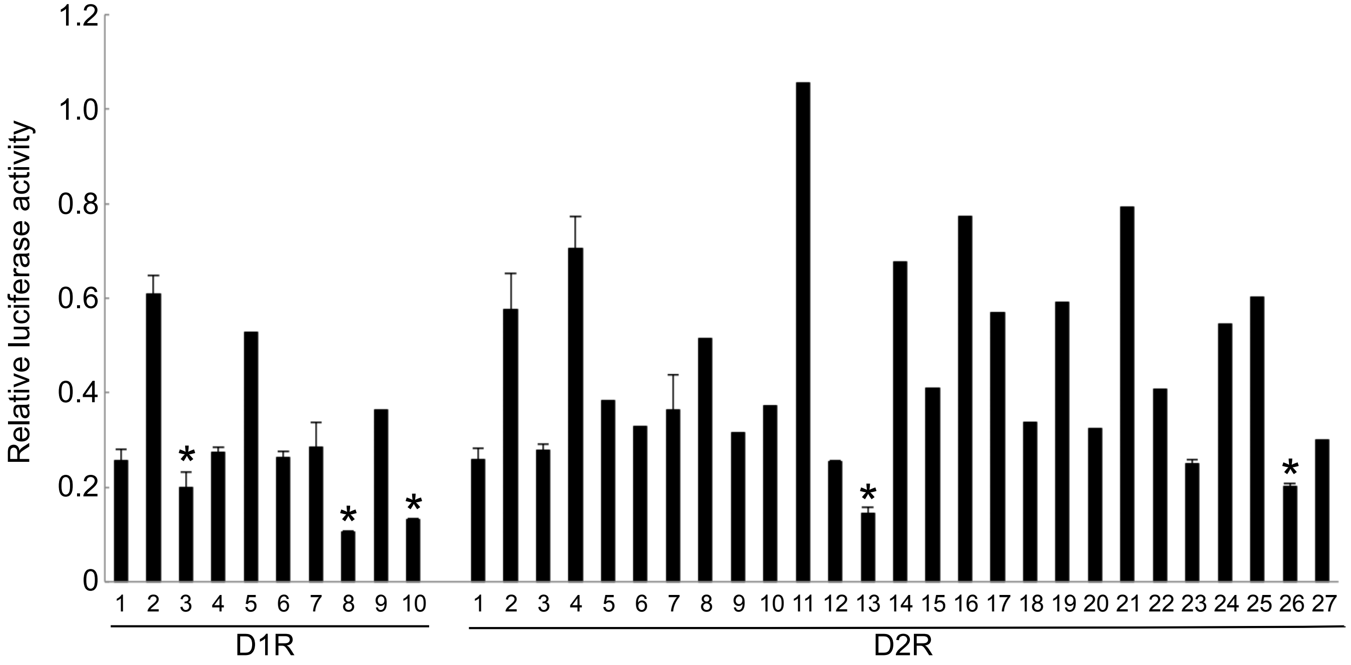

Supplementary Figure 1
Evaluation of silencing efficacy.
Relative ratios of luciferase activity are shown for each shRNA targeting D1R and D2R to the mock cells. The assay system is described in the **METHODS**. The shRNAs (shD1R 1–10 and shD2R 1–27) demonstrating more than 80% knockdown efficacy (labeled with *) are D1R-3 (80%), D1R-8 (89%), D1R-10 (87%), and D2R-13 (86%), and D2R-26 (80%).

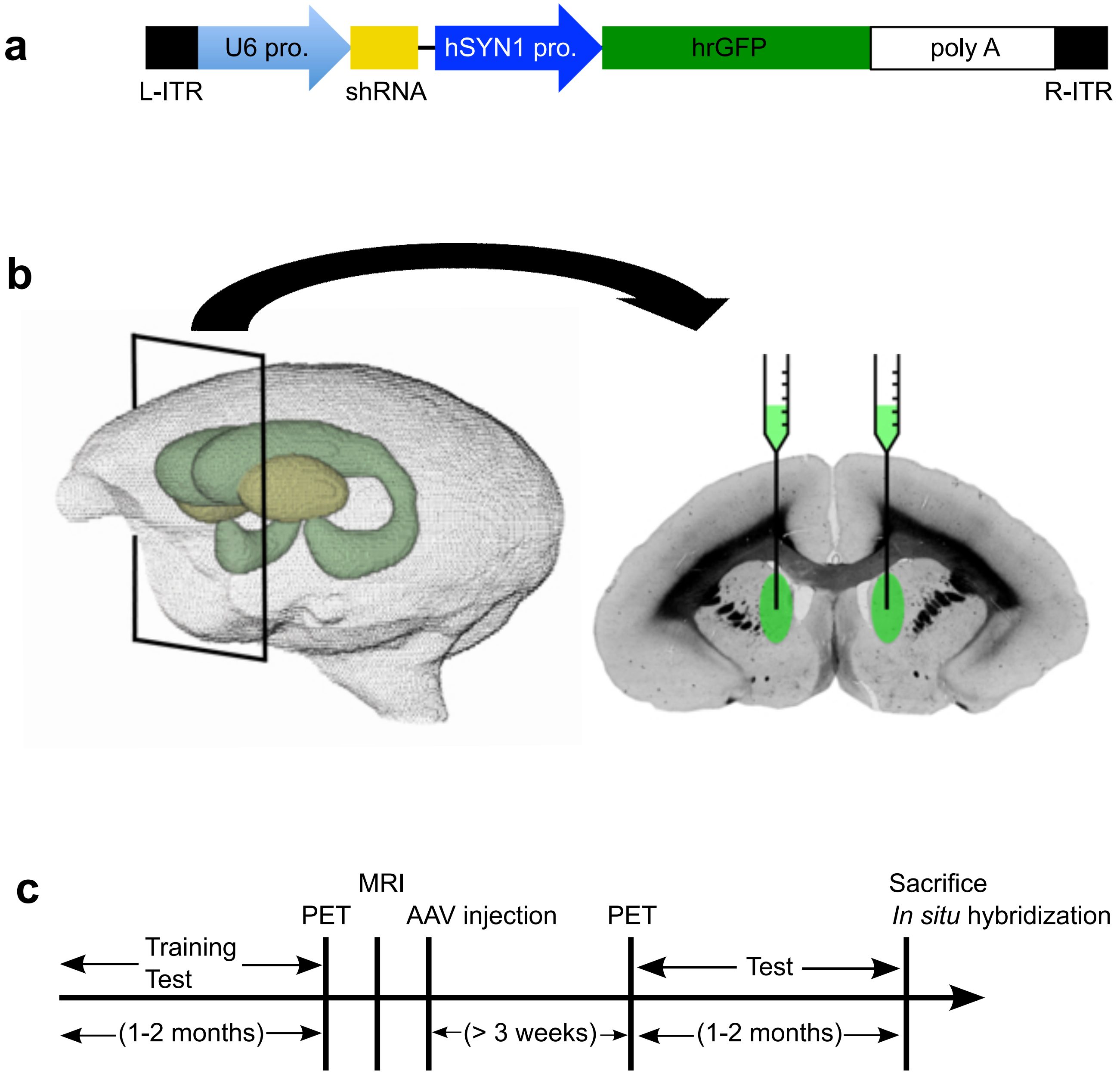

Supplementary Figure 2

Construction and injection of AAV2-mediated D1R-shRNA and D2R-shRNA.

(**a**) Schematic representation of the AAV2 vector construct containing transcriptional units for the U6 promoter-driven shRNA specific for D1R or D2R mRNA and for the hSYN1 promoter-driven hrGFP.

(**b**) Schematic representation of the marmoset caudate nucleus (pale green) and putamen (pale yellow) reconstructed from scanned MR images. AAV2-shRNAs were injected bilaterally into the caudate nucleus.

(**c**) Time course of experimental procedures. Marmosets were trained as described in the legend to **Figure 3** and in the **METHODS**. After the behavioral training sessions were completed, PET images were obtained followed by MRI and vector injections. At least 3 weeks after shRNA injections, PET images were again captured and the behavioral test sessions were conducted as described in **Figure 3**. Analysis of the results from PET scans of D1R KD and D2R KD marmosets were conducted as described in **Figure 1a** and **2a**. After all the experiments were performed, *in situ* hybridization was conducted using postmortem tissue (results shown in **Fig. 1**, **Fig. 2**, and **Supplementary Fig. 3**).

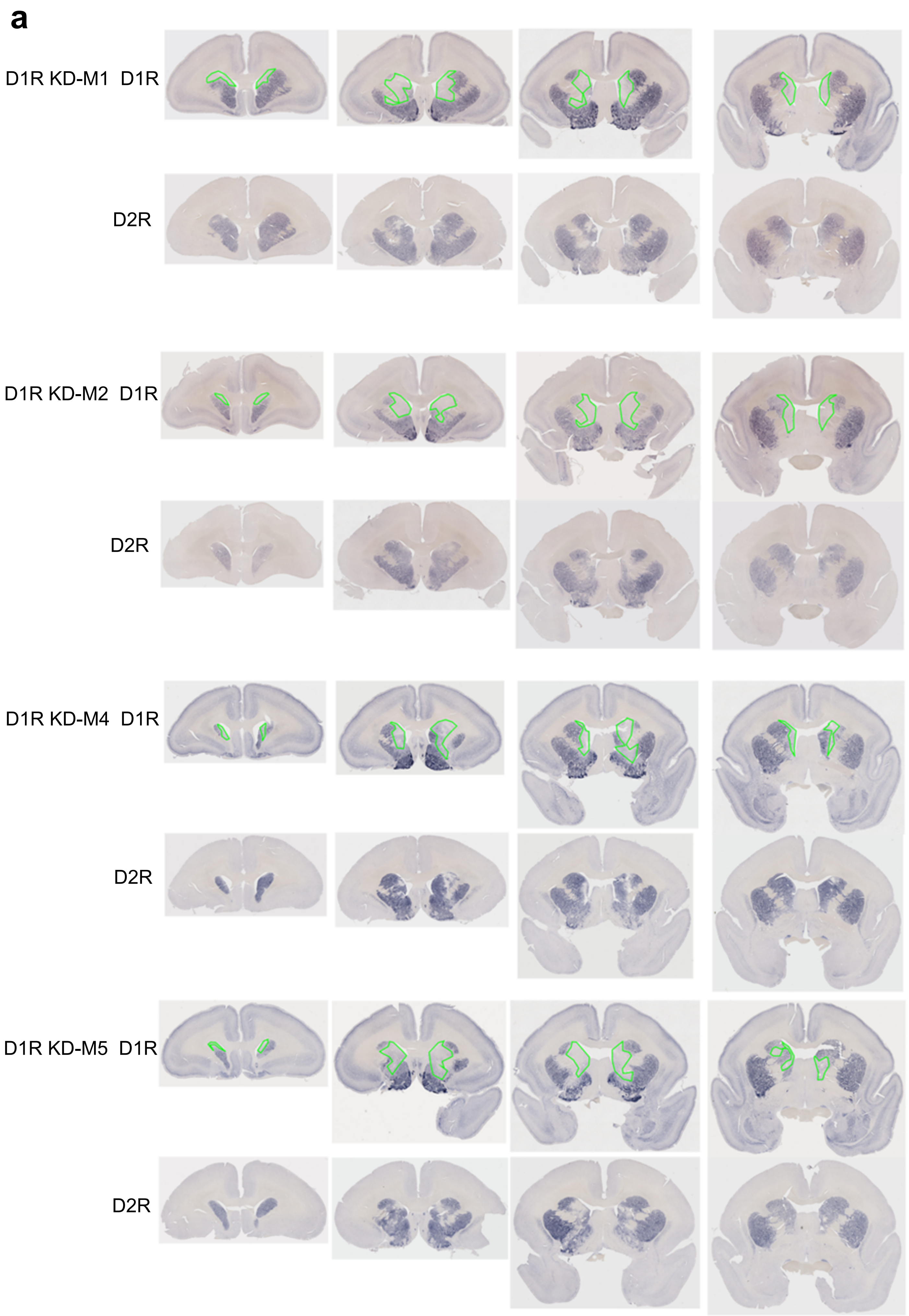

a
D1R KD-M1 D1R
D2R
D1R KD-M2 D1R
D2R
D1R KD-M4 D1R
D2R
D1R KD-M5 D1R
D2R

b

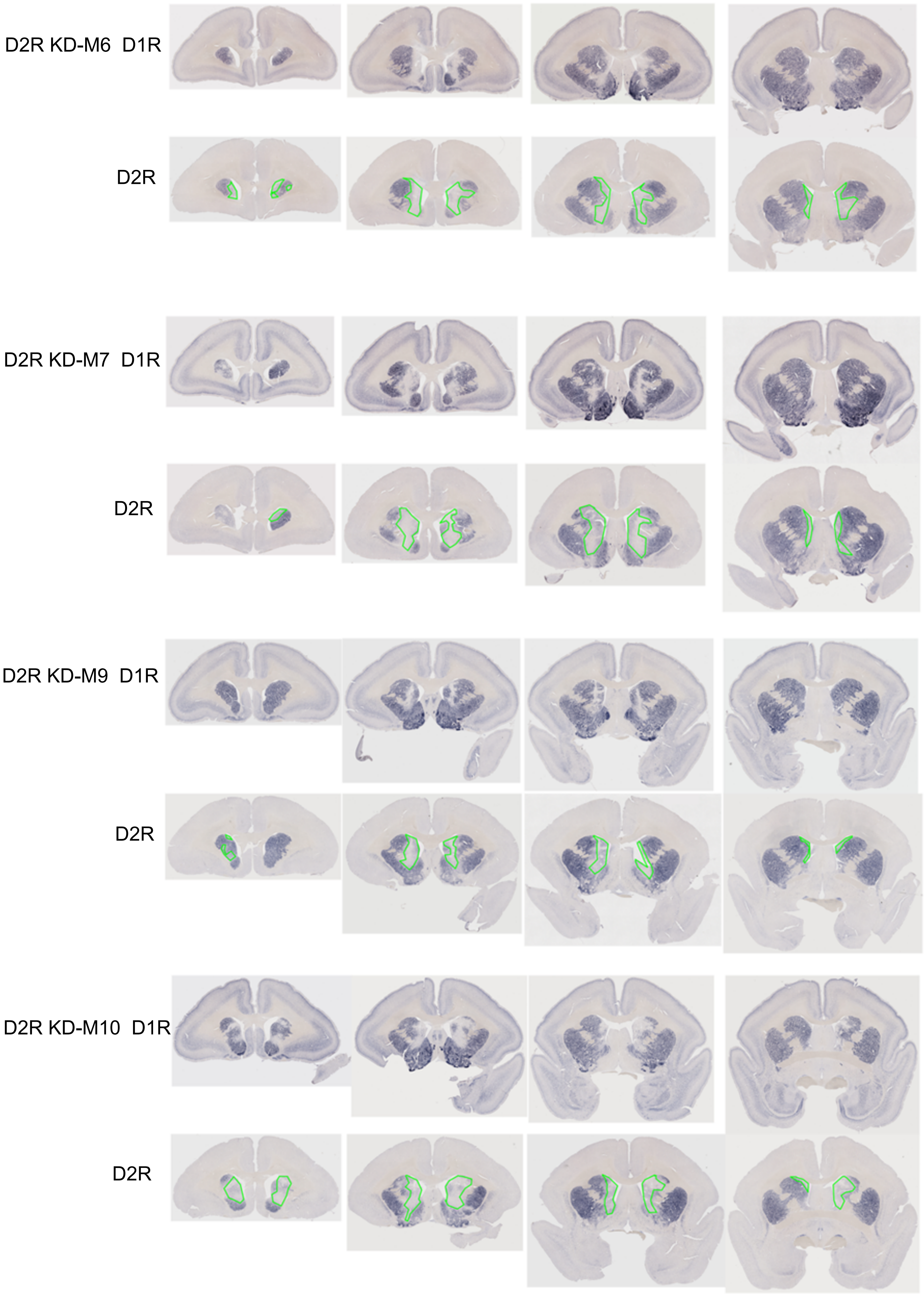

D2R KD-M6 D1R
D2R
D2R KD-M7 D1R
D2R
D2R KD-M9 D1R
D2R
D2R KD-M10 D1R
D2R

Supplementary Figure 3

***In situ* hybridization analyses of D1R and D2R KD marmosets.**

The *in situ* hybridization analyses of four D1R KD marmosets (**a**: M1, M2, M4, M5) and four D2R KD marmosets (**b**: M6, M7, M9, M10) are shown at four different coronal levels. Areas outlined in green show significant reduction in mRNA expression level.

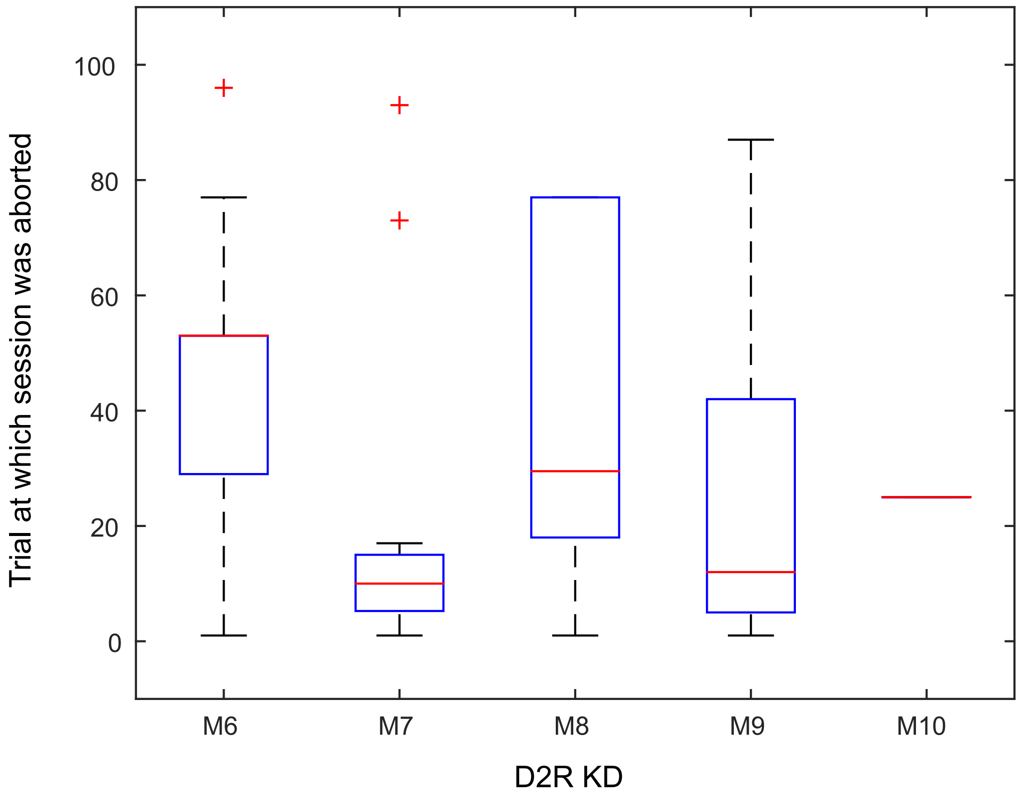

Supplementary Figure 4

Individual difference of D2R KD marmosets in the trial number at which the session was aborted.
Each box plot shows the results of the number of trials in which the session was ceased (aborted) because the animal did not touch the screen for 15 min in after (post) injected D2R KD marmosets. In each box plot, the center red lines represent the medians, the lower and upper edges of the blue boxes indicate the 25th and 75th percentiles, black lines (whiskers) show the minimum and maximum data except for the outliers, and the red crosses show the outliers.

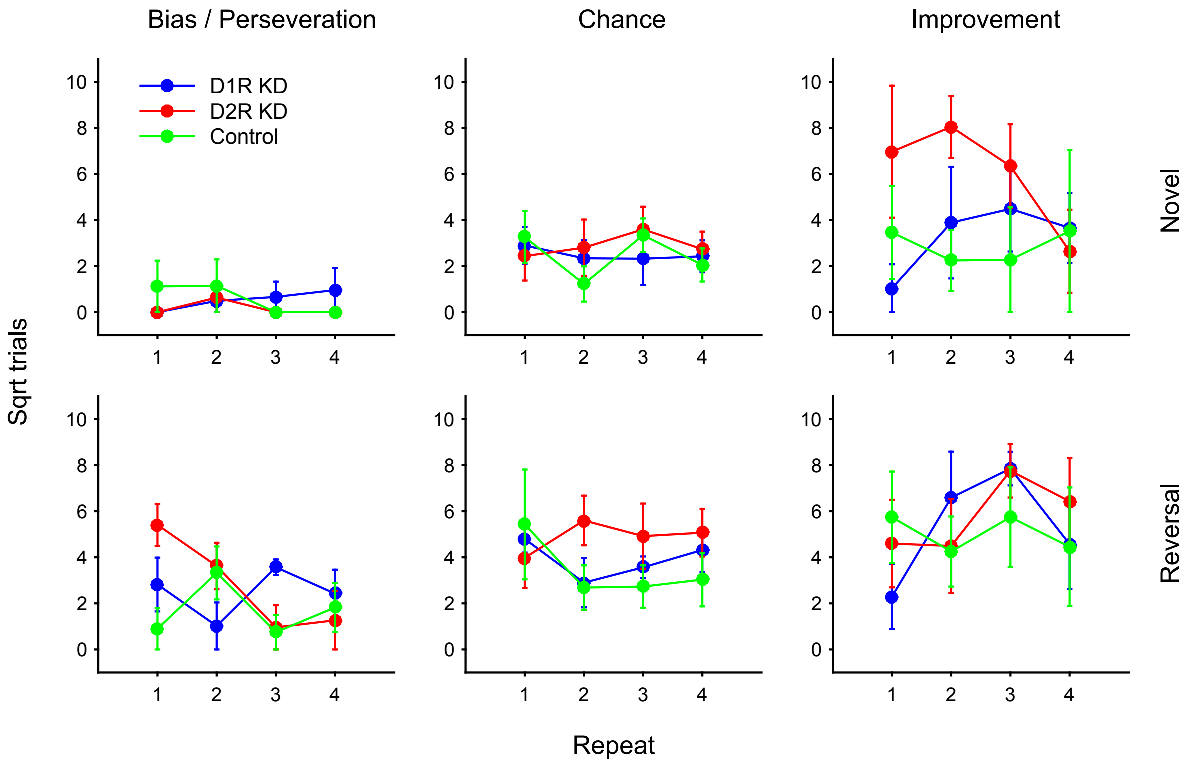

Supplementary Figure 5

Comparison of the number of trials at three stages of learning.

The same data as in **Figure 4** are divided into four repeats of normal (N3–N7) and reversal (R3–R7) learning at three stages of Bias/ Perseveration, Chance, and Improvement (see Text for details). The error bars represent SEM (±).

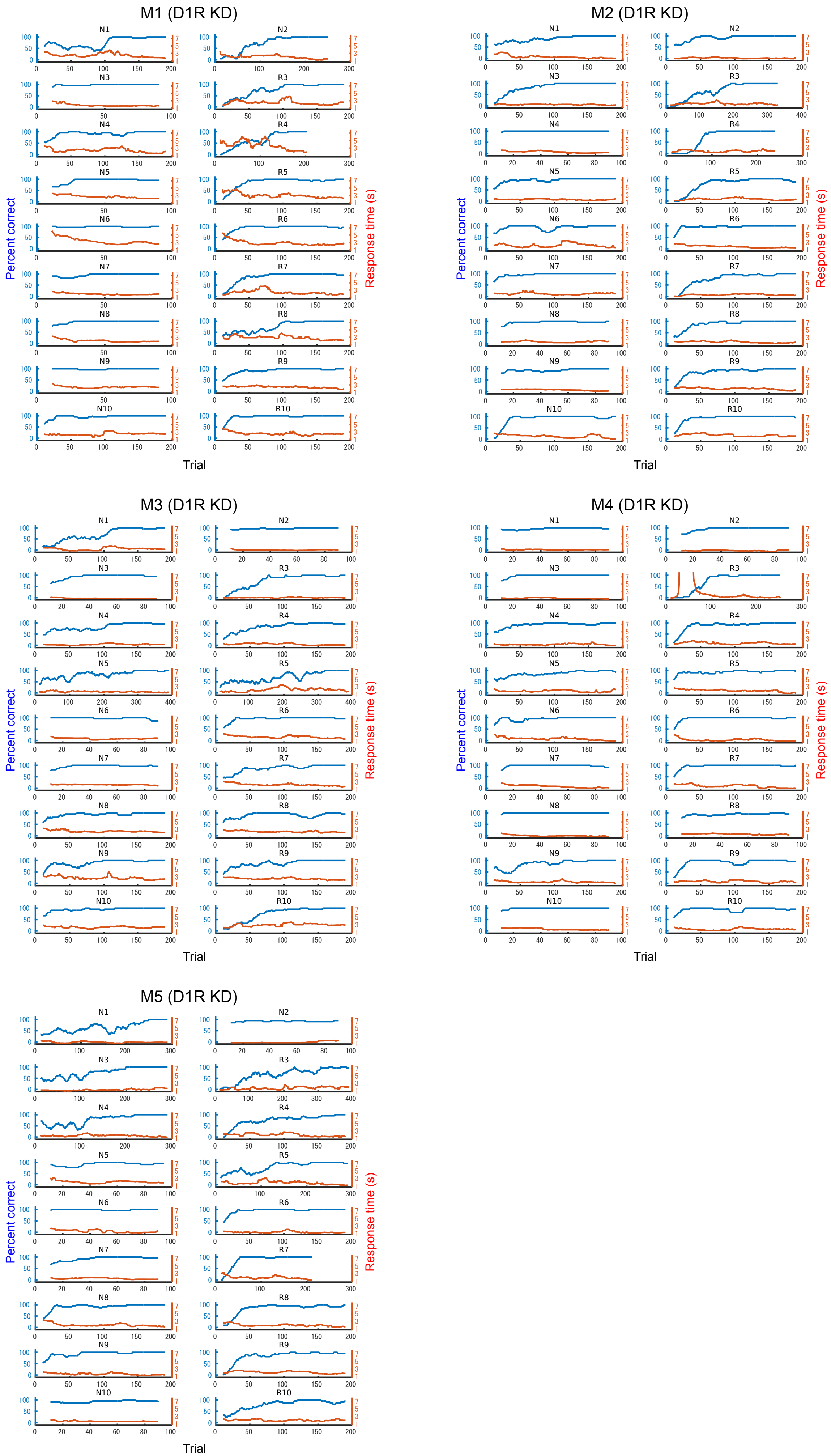

M1 (D1R KD)
M2 (D1R KD)
M3 (D1R KD)
M4 (D1R KD)
M5 (D1R KD)
Percent correct
Response time (s)
Trial
N1
N2
N3
N4
N5
N6
N7
N8
N9
N10
R3
R4
R5
R6
R7
R8
R9
R10

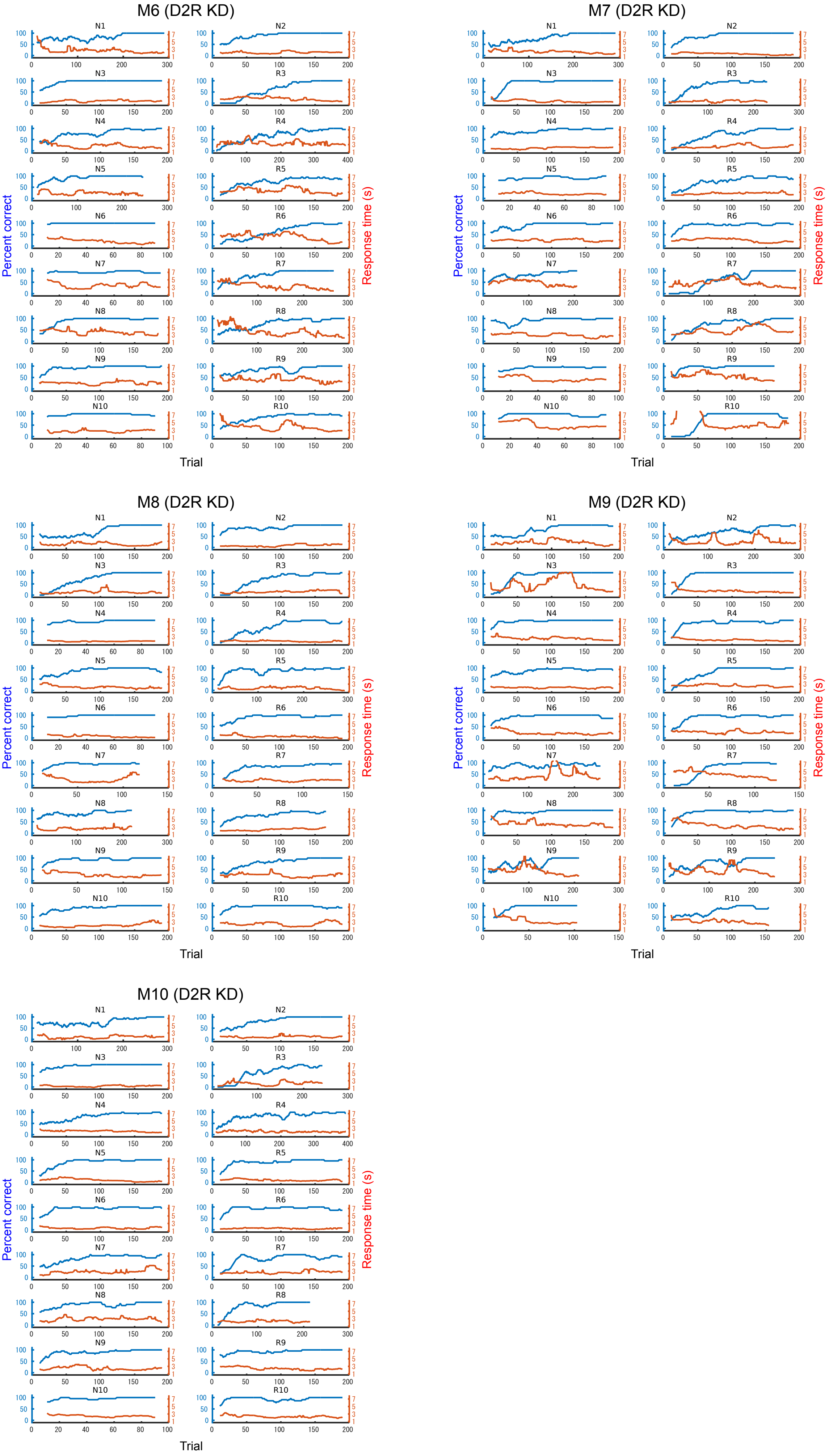
M6 (D2R KD)
M7 (D2R KD)
M8 (D2R KD)
M9 (D2R KD)
M10 (D2R KD)
N1
N2
N3
R3
N4
R4
N5
R5
N6
R6
N7
R7
N8
R8
N9
R9
N10
R10
Percent correct
Response time (s)
Trial

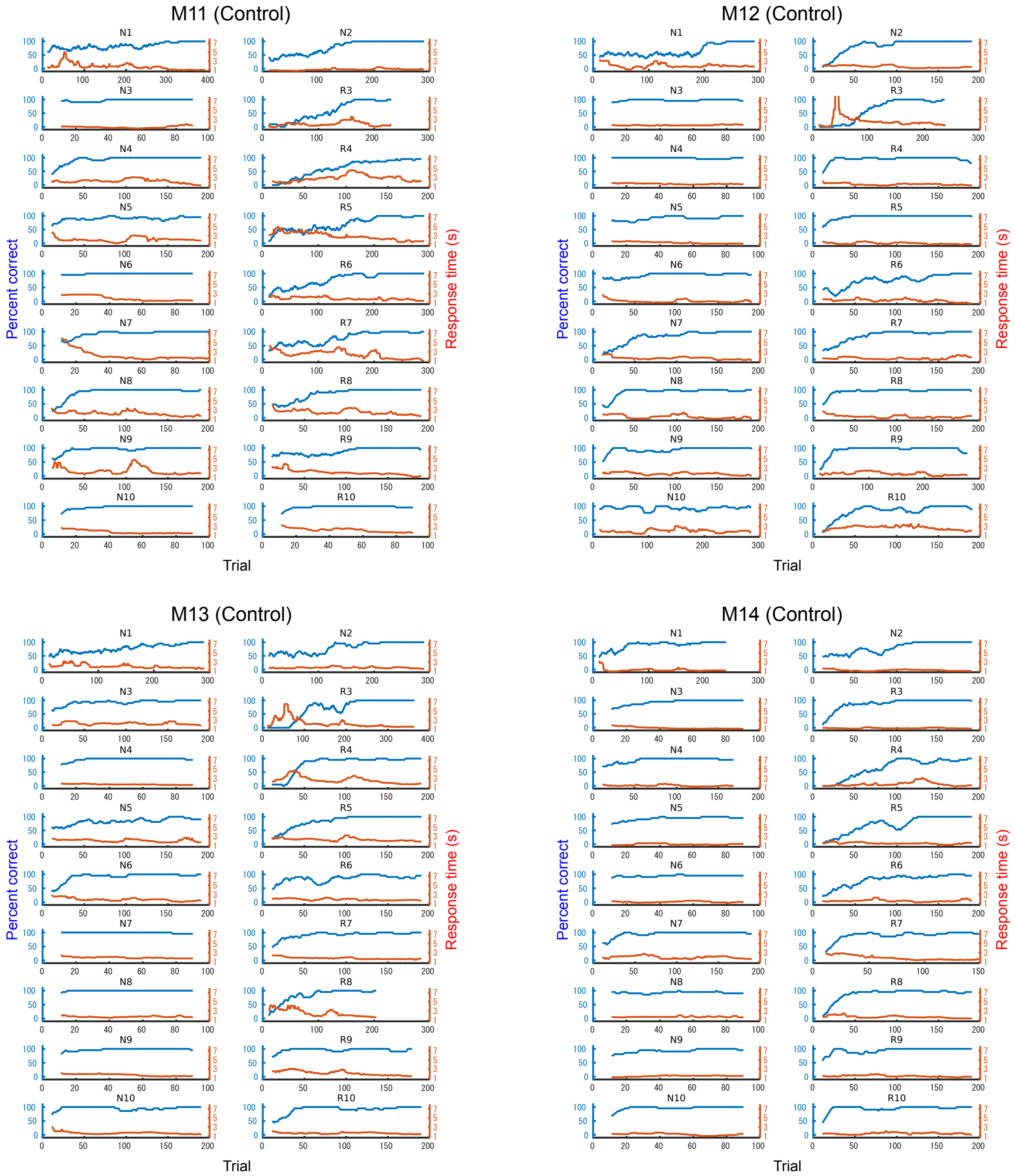
M11 (Control)
M12 (Control)
M13 (Control)
M14 (Control)
N1
N2
N3
N4
N5
N6
N7
N8
N9
N10
R3
R4
R5
R6
R7
R8
R9
R10
Percent correct
Response time (s)
Trial

Supplementary Figure 6

Percent correct and response time of each marmoset for each experiment.

Percent correct (blue line) as a function of trial number was derived with a moving-average method. Response time (red line) was smoothed with a moving–median method. For both the cases, the window length was 21 trials (±10 trials).

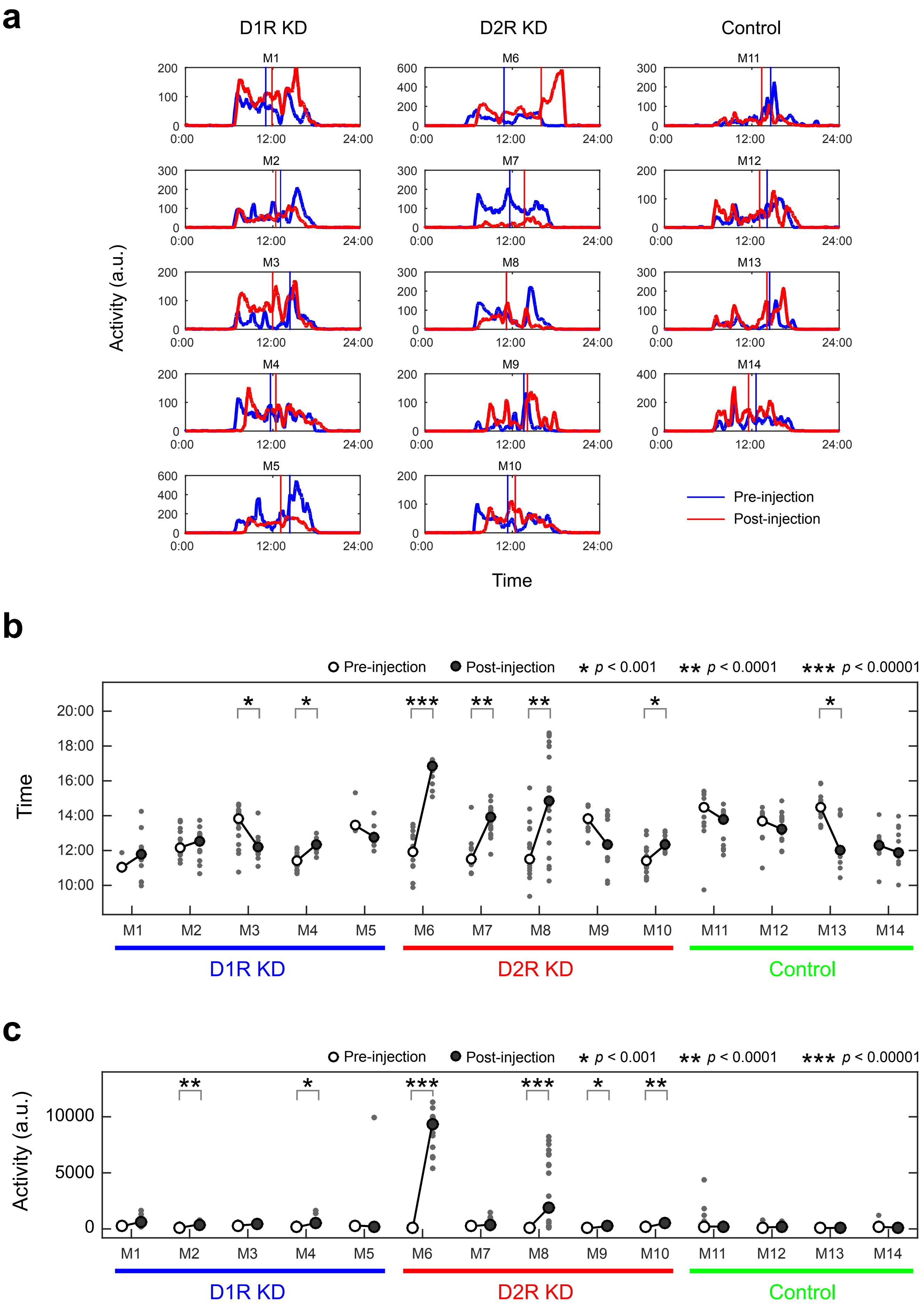
a
D1R KD
D2R KD
Control
M1
M2
M3
M4
M5
M6
M7
M8
M9
M10
M11
M12
M13
M14
Activity (a.u.)
Time
Pre-injection
Post-injection
b
Pre-injection
Post-injection
* p < 0.001
** p < 0.0001
*** p < 0.00001
Time
D1R KD
D2R KD
Control
c
Pre-injection
Post-injection
* p < 0.001
** p < 0.0001
*** p < 0.00001
Activity (a.u.)
D1R KD
D2R KD
Control

Supplementary Figure 7
Locomotor activity before and after AAV2-shRNA injections.
(**a**) Actigrams recorded from each of all 14 marmosets are shown. The ordinate of each graph indicates locomotor activity in an arbitrary unit, and the abscissa represents the time of day. The blue and red curves show averaged activities in 3 consecutive experimental days just before and after the injection, respectively. The data were smoothed using the moving-average method with the 30-min window. The vertical lines represent the central time of daily activity, which is defined as the time point that divides the area under the activity curve into 50 percent. Note that the blue line is overlapped with the red line and not seen in M8.
(**b**) Comparison of locomotor activity between before and after AAV2-shRNA injection. Locomotor activity was compared using Mann-Whitney's U-test. The statistical significance is shown as $^*P < 0.001$, $^{**}P < 0.0001$, $^{***}P < 0.00001$. Each grey point in the figure represents the weighed center of daily activity. Filled circles represent the median.
(**c**) Comparison of locomotor activity between before and after AAV2-shRNA injection for 3 hours after the onset of dark. The ordinate of each graph indicates locomotor activity in an arbitrary unit. Locomotor activity was compared using Mann-Whitney's U-test. The statistical significance is shown as the same as (**b**). Each grey point in the figure represents the 3-hour activity in the dark on each day. Filled circles represent the median.

Supplementary Table 1

Peak voxels obtained from the regression analysis of ^{11}C-raclopride binding with visual discrimination learning performance

Behavioral variable	Direction of relationship	Hemisphere	(x, y, z) Standard space*1 mm	Maximum Z*2
Novel learning errors to criterion	Negative	Left	(-1.6, 0.4, 1.0)	3.35
		Right	(0.8, -1.4, 1.6)	2.25
Reversal learning errors to criterion	Negative	Left	(-1.6, 1.8, 1.0)	3.63
		Right	(3.2, 1.8, -0.2)	3.20
Number of aborted sessions	Negative	Left	(-1.6, 0.6, 1.0)	3.94
		Right	(3.2, 1.6, 0.0)	3.29

*1Coordinates refer to the anterior commissure on the AC-PC plane in the template MR image of the common marmoset brain.

*2Uncorrected $P < 0.05$
